# Oncogenic Kras-Mediated Cytokine CCL15 Regulates Pancreatic Cancer Cell Migration and Invasion through ROS

**DOI:** 10.3390/cancers14092153

**Published:** 2022-04-26

**Authors:** Justin K. Messex, Kiyah L. A. Adams, William G. Hawkins, David DeNardo, Nabeel Bardeesy, Daniel D. Billadeau, Geou-Yarh Liou

**Affiliations:** 1 Center for Cancer Research and Therapeutic Development, Clark Atlanta University, Atlanta, GA 30314, USA; jmessex@cau.edu; 2 Department of Biological Sciences, Clark Atlanta University, Atlanta, GA 30314, USA; kiyah.adams@students.cau.edu; 3 Department of Surgery, Washington University School of Medicine, St. Louis, MO 63110, USA; hawkinsw@wustl.edu; 4 Department of Medicine, Washington University School of Medicine, St. Louis, MO 63110, USA; ddenardo@wustl.edu; 5 Massachusetts General Hospital Cancer Center and Department of Medicine, Harvard Medical School, Boston, MA 02115, USA; nelbardeesy@partners.org; 6 Division of Oncology Research and Schulze Center for Novel Therapeutics, Mayo Clinic, Rochester, MN 55902, USA; billadeau.daniel@mayo.edu

**Keywords:** CCL15, Kras^G12D^, pancreatic ductal adenocarcinoma, cell migration, cell invasion, metastasis, reactive oxygen species

## Abstract

**Simple Summary:**

Oncogenic Kras^G12D^ and tumor inflammation are critical components of the development and dissemination of pancreatic ductal adenocarcinoma (PDAC). The aim of this study is to investigate a lesser-known cytokine, CCL15, that functions as a new downstream target of Kras^G12D^ with the purpose of regulating PDAC cell migration and invasion. We showed increased levels of CCL15 as well as the presence of its receptors, including CCR1 and CCR3, in PDAC tissues and cell lines. The knockdown of CCL15 diminished metastatic Panc-1 cell migration, whereas the treatment of CCL15 in non-metastatic BxPC-3 cells promoted BxPC-3 cell motility. Similar results were verified using murine metastatic PDAC KP-2 cells. Furthermore, we demonstrated that CCL15-modulated PDAC cell migration through the upregulation of cellular reactive oxygen species (ROS) levels and the knockdown of Kras^G12D^ resulted in a decrease in CCL15. Altogether, our data unveiled a new mechanism of oncogenic Kras^G12D^ in modulating PDAC inflammation and spreading.

**Abstract:**

Pancreatic ductal adenocarcinoma (PDAC) is well known for its high death rate due to prompt cancer metastasis caused by cancer cell migration and invasion within the early stages of its development. Here, we reveal a new function of cytokine CCL15, namely the upregulation of PDAC cell migration and invasion. We showed increased levels of CCL15 transcripts and protein expressions in human PDAC tissue samples, as well as in cultured cell lines. Furthermore, PDAC cells also expressed CCL15 receptors, including CCR1 and CCR3. Murine PDAC cell lines and tissues strengthened this finding. The manipulation of CCL15 in metastatic Panc-1 cells through CCL15 knockdown or CCL15 neutralization decreased Panc-1 cell motility and invasiveness. In addition, treating non-metastatic BxPC-3 cells with recombinant CCL15 accelerated the cell migration of BxPC-3. A reduction in the levels of reactive oxygen species (ROS) by either N-Acetyl-L-Cysteine treatment or p22phox knockdown led to a decrease in Panc-1 cell migration and a reversed effect on recombinant CCL15-promoted BxPC-3 cell movement. Importantly, the knockdown of oncogenic Kras in Panc-1 cells abolished CCL15 protein expression and impeded cell migration without affecting PDAC cell growth. Altogether, our work elucidates an additional molecular pathway of oncogenic Kras to promote PDAC metastasis through the upregulation of cell migration and invasion by the Kras downstream CCL15, a lesser-known cytokine within the cancer research field.

## 1. Introduction

Pancreatic ductal adenocarcinoma (PDAC) is one of the most lethal types of cancer, with a dismal five-year survival rate of around 7%. Late diagnosis in clinical settings is common, as is the early metastasis of cancer cells to other parts of the body, limiting therapeutic options and contributing to the high death rate. PDAC is a disorder of oncogenic Kras, a characteristic that is substantiated by the 90% of patients with PDAC harboring oncogenic Kras mutations. Furthermore, turning off Kras mutants in transgenic PDAC mice reversed cancer cells back to normal pancreatic acinar cells [1,2,3]. Initially, cancer metastasis involves an increase in cancer cell migration and invasion that allows local cancer cells to physically leave the original site. To maintain their increased migration and mobile abilities, cancer cells undergo epithelial-mesenchymal transition (EMT) to alter the cell–cell adhesion and the apical–basal polarity [4,5,6,7]. Key molecules positively associated with EMT, such as YAP, TAZ, and Snail, have been detected in PDAC [8,9,10]. 

Cytokine CCL15 has been reported to play a role in asthma through IgE-regulated immunomodulation since high levels of CCL15 detected in the serum samples of patients with severe asthma can be diminished by a humanized anti-IgE antibody [11,12]. However, so far, very little is known about this cytokine as it pertains to the cancer research field. The most well-known function of CCL15 is its chemotactic ability to attract neutrophils, monocytes, and lymphocytes to re-shape the tumor microenvironment [10,13]. Although a few reports showed that overexpressed CCL15 in cancer cells may be used as an indicator of poor patient outcomes of hepatocellular carcinoma and colorectal cancer metastasis [14,15,16,17], the mechanisms used by CCL15 within cancer cells to regulate their metastatic abilities remain unclear.

We, herein, showed that increased expressions of CCL15 were detected in human and murine metastatic PDAC cells. Results from the TCGA Genomic database suggested that patients with PDAC with the CCL15 copy number amplification had poor clinical outcomes as compared to those without the amplification. Both CCR1 and CCR3, which are receptors for CCL15, were present in PDAC cells. A blockade of CCL15 in human metastatic PDAC Panc-1 cells, either through CCL15 knockdown or CCL15 neutralization, significantly impeded cancer cell motility as well as invasion. Similarly, the knockdown of CCL9, an orthologue of human CCL15 in murine metastatic PDAC KP-2 cells, attenuated their cell migratory ability. On the other hand, the addition of exogenous recombinant CCL15 to non-metastatic BxPC-3 cells accelerated the cell migration of BxPC-3. Interestingly, treating human PDAC cells with a general ROS scavenger, NAC, reduced the CCL15-mediated cell migration of PDAC. Furthermore, the knockdown of Kras in human Panc-1 cells suppressed CCL15 protein expression, suggesting that CCL15 functions downstream of oncogenic KRas^G12D^ in PDAC. In summary, our data not only unveil a new function of CCL15, which is to regulate PDAC cell migration and invasion, but also elucidates another new mechanistic regulation that oncogenic Kras mutants use to promote PDAC cell metastasis through CCL15 and its subsequent ROS production.

## 2. Materials and Methods

### 2.1. Cell Lines, Antibodies, and Reagents

Human pancreatic ductal adenocarcinoma (PDAC) cell lines, including BxPC-3, Panc-1, and CFPAC-1, were obtained from ATCC (Manassas, VA, USA) and cultured according to the instructions of ATCC. Murine pancreatic ductal cells GA36 were derived from six-week-old Pdx1^cre/+^/Kras^G12D/+^ mice as previously described [18], cultured on collagen I-coated dishes, and maintained in DMEM/F-12 media with 15 mM HEPES with supplements of 5 mg/mL D-glucose, 1.22 mg/mL nicotinamide, 5 nM 3,3,5-tri-iodo-L-thyronine, 1 µM dexamethasone, 100 ng/mL cholera toxin, 5 mL/L insulin-transferrin-selenium, 0.1 mg/mL soybean trypsin inhibitor type I, 20 ng/mL EGF, 5% NuSerum IV, and 25 µg/mL bovine pituitary extract. Murine PDAC cells KP2 were cultured on collagen I-coated plates and maintained as previously described [19,20]. All cells were maintained in a 37 °C incubator with 5% CO_2_. CCL15 antibody was purchased from Abcam (Cambridge, UK). Both CCR1 and CCR3 antibodies were from Novus Biologicals (Littleton, CO, USA). N-cadherin antibody was purchased from Thermo Fisher Scientific (Waltham, MA, USA) and antibodies of vimentin and p22phox were from Cell Signal Technology (Danvers, MA, USA). Antibodies of actin and GAPDH were from Santa Cruz Biotechnology (Dallas, TX, USA). Kras antibody was purchased from ProteinTech (Rosemont, IL, USA). Recombinant CCL15- and CCL15-neutralizing antibodies were from PeproTech (Cranbury, NJ, USA) and R&D Systems (Minneapolis, MN, USA), respectively. N-Acetyl Cysteine was purchased from Sigma-Aldrich (St. Louis, MO, USA). Other reagents used are described in the specific experiment sections.

### 2.2. Gene Knockdown

Lentiviral plasmids encoding shKras, shCCL15, shCCL9, and scramble were purchased from Sigma-Aldrich (St. Louis, MO, USA) with the following TRC clone ID numbers. For shKras, TRCN0000040149 (labeled as shKras#1) and TRCN 0000040151 (labeled as shKras#2); for shCCL15, TRCN0000377734 (labeled as shCCL15#1) and TRCN0000371599 (labeled as shCCL15#2); for shCCL9, TRCN0000077010 (labeled as shCCL9#1) and TRCN0000077012 (labeled as shCCL9#2). Lentivirus was produced in 293FT cells that were transfected with the plasmid as described above and ViraPower Packaging Mix (Invitrogen; Waltham, MA, USA) using Lipofectamine 2000, and lentiviral titers were determined according to the manufacturer’s instructions. Cells were infected with shKras, shCCL15, shCCL9, or shScramble lentiviruses in the presence of 6 µg/mL fresh polybrene for 24 h. After 24 h, the media containing lentiviruses were removed and replenished with fresh complete media. 72 h post-infection, fresh complete media containing appropriate antibiotics were added to select knockdown cells and changed every 2–3 days. One week after antibiotic selection, cells in pool were collected for real-time quantitative RT-PCR or immunoblotting to assess gene knockdown efficiency.

### 2.3. Cell Lysates Collection and Immunoblotting

Cells were rinsed twice with cold PBS, lysed in buffer A (50 mM Tris/HCl [pH 7.4], 1% TritonX-100, 150 mM NaCl, and 5 mM EDTA [pH 7.4]) or RIPA buffer (25 mM Tris/HCl [pH 7.5], 1% Triton X-100, 140 mM NaCl, 1 mM EDTA, 0.5% SDS) supplemented with a protease inhibitor cocktail (Thermo Fisher Scientific), vortexed with the maximal speed for 1 min, incubated on ice for 10 min, and centrifuged at 4 °C, 14,000 rpm for 10 min. The supernatants were denatured, followed by SDS-PAGE. Resolved proteins were transferred onto nitrocellulose membranes followed by blocking in 5% BSA in TBST (50 mM Tris.HCl [pH 7.6], 150 mM NaCl, 0.05% Tween 20) and incubated with the antibodies of interest in 5% BSA in TBST overnight at 4 °C. The appropriate horseradish peroxidase-conjugated secondary antibodies were added to the membranes for 30 min at room temperature. Images were captured and visualized with ECL and X-ray film.

### 2.4. RNA Isolation and Real-Time Quantitative PCR

Total RNA was isolated from cells using the RNeasy kit from Qiagen (Hilden, Germany) according to the manufacturer’s instructions. Levels of mRNA of interest were examined using a 2-step quantitative reverse transcriptase-mediated real-time PCR (qPCR) method. An equal amount of total RNA was converted to cDNA by the high-capacity cDNA reverse transcriptase kit (Applied Biosystems, Bedford, MA, USA). Real-time quantitative PCR was performed in a CFX Connect real-time PCR detection system (Bio-Rad) using the TaqMan Universal PCR master mix (Applied Biosystems) with probe/primer sets and the following PCR program: 95 °C for 20 s, 40 cycles of 95 °C for one second, and 60 °C for 20 s. All Taqman probe/primer sets were purchased from Applied Biosystems (CCL15: Mm01302419_m1; CCL9: Mm00441260_m1; GAPDH: Mm99999915_g1 for mouse and Hs99999905_m1 for human). The collected data were analyzed by Sequence Detection System software (Bio-Rad) and normalized to GAPDH. The mRNA abundance was calculated using the ΔΔ*C*_T_ method.

### 2.5. Immunohistochemistry

Human pancreatic tissue slides, including normal and cancer tissue samples, were obtained from BioChain (Newark, CA, USA) and US Biomax (Derwood, MD, USA). All experiments that involved using human tissue samples were carried out according to the IRB protocol approved by the CAU IRB committee. Slides were deparaffinized in xylene and gradually re-hydrated through 100% alcohol to distilled water. The re-hydrated slides were subjected to heat-induced antigen retrieval in the antigen retrieval buffer, 10 mM sodium citrate buffer (pH 6.0) or 10 mM Tris. Slides were then incubated with 3% hydrogen peroxide followed by PBS wash. Slides were treated with protein block serum-free solution (DAKO) for 10 min at room temperature. After the primary antibody (CCL15 1:400 (Abcam); CCR1 1:100 (Novus Biologicals); CCR3 1: 200 (Abcam)) was incubated with slides, the ImmPRESS Polymer Detection Kit (Vector Laboratories; Burlingame, CA, USA) was used according to the manufacturer’s instructions. Images were collected using the Aperio VERSA tissue scanner with ImageScope software (Aperio; Sausalito, CA, USA). For specificity of CCR1 and CCR3 antibody, see Figure S10.

### 2.6. Measurement of Cellular ROS Levels

1 × 10^4^ cells/well of BxPC-3 or Panc-1 cells were seeded in a 96 black well plate with a clear bottom. The following day, the cells were labeled with 20 µM H_2_DFFDA in complete media at 37 °C for 20 min. Cells were then washed 3 times with pre-warmed Live Cell Imaging Solution (Thermo Fisher Scientific). After the final wash, cellular ROS levels were measured in Live Cell Imaging Solution using the Synergy H1 Hybrid Microplate Reader (Agilent Technologies; Santa Clara, CA, USA) at 485/528 (Ex/Em) nm. For treatments of recombinant CCL15 (100 ng/mL) or CCL15 neutralizing antibody (NAb) (5 µg/mL), cells were treated for 24 h before H_2_DFFDA labeling and subsequent cellular ROS measurement as described above.

### 2.7. Wound Healing Assay/Cell Migration Assay

6 × 10^4^ BxPC-3 or Panc-1 cells were seeded in each well of the 2-well culture-insert placed in a 24-well plate and allowed to reach confluence prior to the removal of the culture-inserts. The cells were then washed with PBS twice, and appropriate growth media containing 1% FBS was carefully added to the well to allow cells to migrate into the gap caused by the culture-insert (set as 0 h). For testing the recombinant CCL15 effect on BxPC-3 migration, 1 × 10^5^ BxPC-3 cells were used in the described wound healing assay. For KP2 cell migration, 1 × 10^5^ cells were seeded in each well of the 2-well culture-insert into a collagen I-coated 24-well plate used in the procedure described above. Images were captured using a Zeiss Axiovert 200M inverted microscope with a 10× objective lens at the indicated time points. The area of the culture-insert gap was recorded. At each indicated time point, the area of the gap was measured using image J software, while the migrated area was calculated based on the difference between the area recorded at t = 0 and the empty area at the indicated time point. The final number was recorded as a percentage of migrated area compared to t = 0, the initial area prior to migration.

### 2.8. Cell Growth Assay

1 × 10^5^ cells were seeded in a 12-well plate in complete media containing puromycin to maintain the knockdown of the indicated gene. Cells were trypsinized, and alive cell numbers were counted using the trypan blue exclusion method every 24 h up to 96 h. Each time point indicated was quantified by three separate wells.

### 2.9. Cell Invasion Assay

The indicated Panc-1 cells were serum starved in the serum-free media for 24 h, detached from cell culture dishes by trypsin/EDTA, counted, and re-suspended in serum-free media containing 0.1% BSA at a density of 1 × 10^6^ cells/mL. A quantity of 750 µL serum-free media containing 0.1% BSA and 1 ng/mL EGF was added to each bottom well of the Corning BioCoat Matrigel Invasion Chamber. The matrigel-coated insert (8 µm pore size) was placed on top of each well in the 24-well Corning BioCoat Chamber plate and 200 µL of cell solution was added to each insert. The whole plate was returned to the cell culture incubator for 16 h. At the end point, the inserts were vigorously washed with PBS twice, and cells on each side of the membrane of the insert were fixed with 3.7% formaldehyde in PBS at room temperature for 2 min. Membranes of the inserts were then washed with PBS twice. Cells on the membrane were permeabilized in 100% methanol at room temperature for 20 min, washed with PBS twice, and stained with Giemsa at room temperature for 15 min in the dark. After another wash with PBS, un-invasive cells that were on the top side of the membrane were removed by cotton swabs. Images of the invaded cells on the bottom of the membrane of the insert were collected using a Zeiss Axiovert 200M inverted microscope with a 20× objective lens. The number of invasive cells was counted in five randomly selected fields per condition.

### 2.10. Statistical Analysis

Data are presented as means ± SE. *p*-values were acquired with the Student’s *t*-test for comparing 2 sets of data using Prism (GraphPad Software, San Diego, CA, USA). For more than 2 sets of data, one-way ANOVA analysis along with multiple comparisons was carried out using Prism (GradPad Software). *p* < 0.05 is considered statistically significant.

## 3. Results

### 3.1. Elevated Expression of CCL15 and Its Receptors in Human PDAC

Based on the results of our microarray experiments, CCL15 was identified as a top-ranked target involved in the cancer progression of PDAC. So far, there is very limited information on CCL15 function within physiological and pathological settings; therefore, we first utilized the TCGA database of Cancer Genomics to assess the role of CCL15 in pancreatic cancer (http://www.cancer.gov/tcga, accessed on 19 April 2022). Among all types of pancreatic cancer, including pancreatic adenocarcinoma, pancreatic neuroendocrine tumors, and undifferentiated pancreatic carcinoma found in the database, which were extracted from 596 patient samples of three PDAC studies, it showed that CCL15 gene amplification was only detected in pancreatic adenocarcinoma (Figure S1A). Although CCL15 showed a low frequency rate of 2.5%, it did suggest specificity to PDAC. Moreover, with the available data, we found that patients with PDAC with CCL15 amplification demonstrated significantly shorter median survival than those without amplification (4.7 vs. 20.2 months, *p* = 0.00184) (Figure S1B). Altogether, these data indicate the importance of CCL15 in human PDAC.

**Figure 1:**
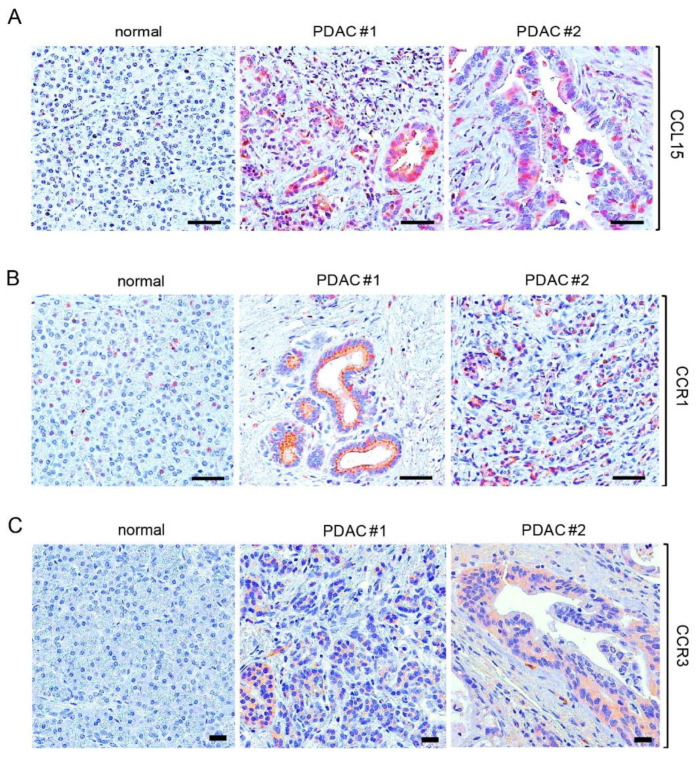
Increased expression of CCL15 and its receptors in human tissue samples of PDAC. (**A**–**C**) Human tissues of normal pancreas and PDAC were immunostained with antibodies of CCL15 (**A**), CCR1 (**B**), and CCR3 (**C**). Scale bar: 50 µm.

To further verify the idea that CCL15 plays a role in PDAC, we evaluated CCL15 expression in human PDAC tissue samples through immunohistochemistry. As shown in Figure 1A, the increased expression of CCL15 was present in human PDAC tissues as compared to the normal human pancreas. It has been reported that CCR1 and CCR3 function as specific receptors for cytokine CCL15 resulting in biological functions through the ligand-receptor interaction [13,21,22]. We also detected the expression of both CCR1 and CCR3 in PDAC cells of human tissue samples (Figure 1B,C).

### 3.2. Upregulation of CCL15 in Metastatic PDAC Cells versus Non-Metastatic PDAC Cells

**Figure 2:**
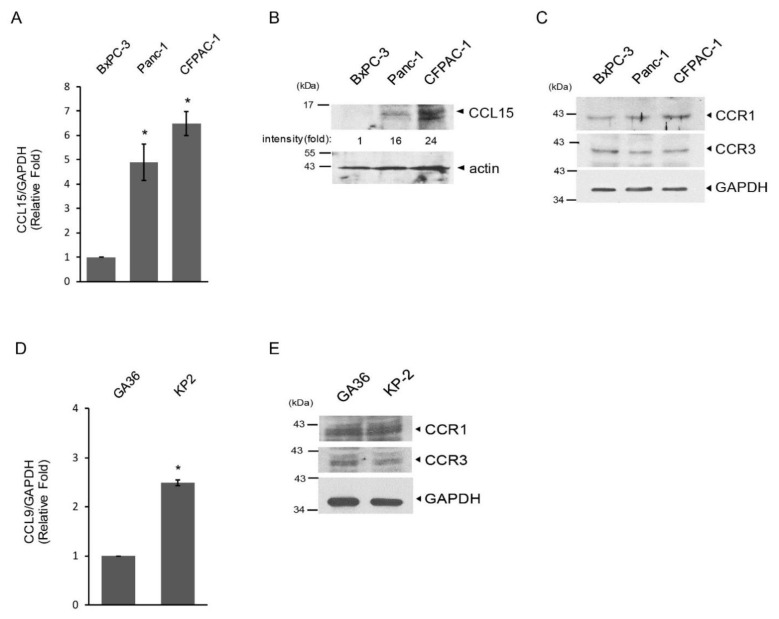
Upregulation of CCL15 levels in metastatic PDAC cells. (**A**) Levels of CCL15 mRNA in human PDAC cell lines (BxPC-3: non-metastatic; Panc-1 and CFPAC-1: metastatic) were assessed by real-time qRT-PCR. Levels of GAPDH were used as an internal control. *: *p* < 0.05 as compared to BxPC-3. (**B**) Levels of CCL15 protein expression in human PDAC cell lines were examined by immunoblots. Levels of actin protein were used as a loading control. (**C**) Protein levels of CCR1 and CCR3 in human PDAC cell lines were evaluated by immunoblots. GAPDH protein levels were used as a loading control. (**D**) mRNA levels of CCL9, the orthologue of CCL15 in murine PDAC cells KP2 and pancreatic ductal cells GA36 were assessed by real-time qRT-PCR. Levels of GAPDH mRNA were used as an internal control. *: *p* < 0.05 as compared to GA36. (**E**) Levels of CCR1 and CCR3 expression in murine GA36 and KP2 cells were determined by immunoblots. GAPDH protein levels were used as a loading control. Original blots see Supplementary File S1.

One of the critical features of PDAC in clinical settings is its early metastasis to other organs, which is highly associated with cancer cell migration and invasion. To assess if CCL15 plays a role in cancer metastasis, we first examined the levels of CCL15 mRNA and protein expression in cultured human PDAC cell lines that have different metastatic abilities when implanted into mice [23,24,25]. These included non-metastatic BxPC-3 and metastatic Panc-1 and CFPAC-1 cells. We detected a higher expression of CCL15 transcripts as well as proteins in Panc-1 and CFPAC-1 cells, both of which are metastatic rather than non-metastatic BxPC-3 cells (Figure 2A,B). In addition, all three human PDAC cell lines expressed the CCL15 receptors CCR1 and CCR3 (Figure 2C). Similarly, the levels of CCL9, an orthologue of human CCL15, were elevated in murine metastatic PDAC KP-2 cells [19,20] as compared to those in murine pancreatic ductal GA36 cells (Figure 2C). Furthermore, the increased expression of CCL9 was detected in metastatic PDAC cells of pdx1^CRE+^/Kras^G12D^/p53^R172H^ (KPC) mice in comparison to adjacent normal acini of the pancreas (Figure S2). Altogether, these data bolster our results obtained from cultured human PDAC cell lines. Finally, both GA 36 and KP-2 cells did express CCR1 and CCR3 (Figure 2E).

### 3.3. CCL15-Modulated PDAC Cell Migration through Reactive Oxygen Species (ROS)

**Figure 3:**
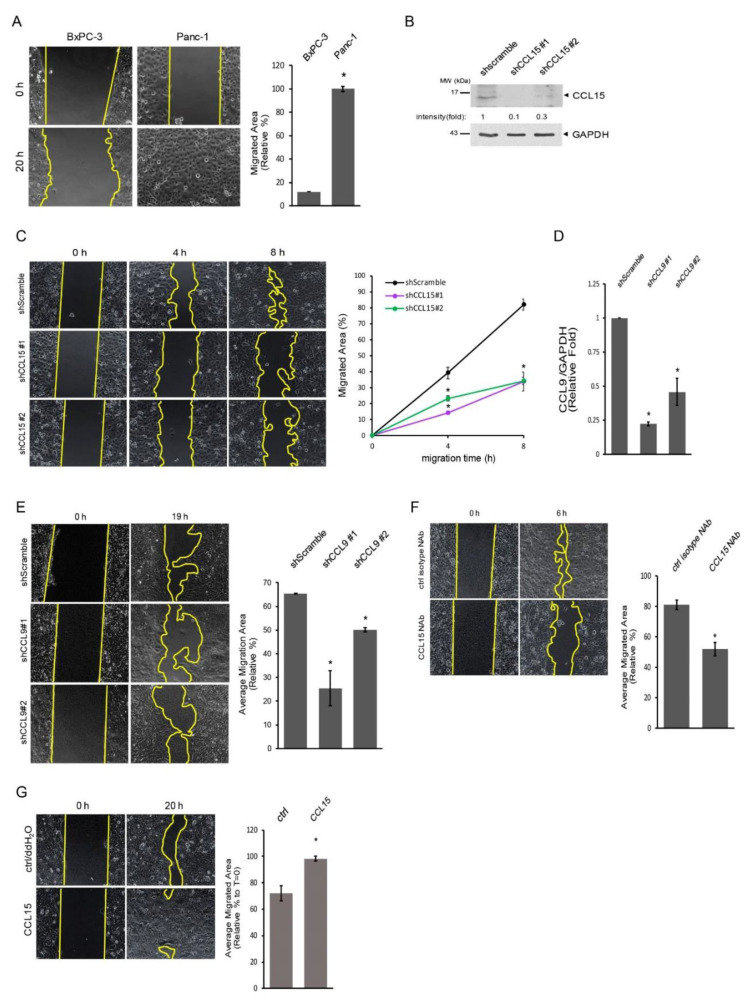
CCL15 mediated PDAC cell migration. (**A**) BxPC-3 and Panc-1 cells were seeded in culture-inserts as described in Section 2. After removal of culture-inserts, cells migrated into the existing gap areas (yellow lines indicate the cell migration front lines) and monitored for 20 h, *: *p* < 0.05. (**B**) Levels of CCL15 in Panc-1 cells stably expressing shScramble, shCCL15 #1, or shCCL15 #2 were assessed by immunoblots. GAPDH levels were used as a control to assess knockdown efficiency. Quantification result from 4 independent experiments is shown in Figure S3. (**C**) Similar to A, Panc-1 cells stably expressing shScramble, shCCL15 #1, or shCCL15 #2 were assessed for their cell migration abilities at the indicated time points. *: *p* < 0.05 as compared to shScramble at same migration time point. (**D**) Levels of CCL9, an orthologue of human CCL15, in murine PDAC KP2 cells stably expressing shScramble, shCCL9 #1, or shCCL9 #2 were examined by real-time qRT-PCR. *: *p* < 0.05 as compared to shScramble. (**E**) Murine PDAC KP2 cells stably expressing shScramble, shCCL9 #1, or shCCL9 #2 were subjected to a cell migration assay. The cells were allowed to migrate for 19 h after removal of culture-inserts. *: *p* < 0.05 as compared to shScramble. (**F**) Panc-1 cells were treated either with a control isotype neutralizing antibody or a CCL15 NAb (5 µg/mL) followed by cell migration assay. *: *p* < 0.05 as compared to control isotype NAb. (**G**) BxPC-3 cells treated with either ddH_2_O/control or 50 ng/mL recombinant CCL15 were subjected to a cell migration assay for 20 h. *: *p* < 0.05 as compared to control. Original blots see Supplementary File S1.

Next, we assessed the migration ability of metastatic Panc-1 cells and non-metastatic BxPC-3 cells using the wound-healing assay. As expected, Panc-1 cells that contained increased levels of CCL15 migrated faster than BxPC-3 cells, filling in the vacant areas at a quicker rate (Figure 3A). To validate the link between CCL15 and PDAC cell migration, we knocked down CCL15 with two different sequences specifically targeting CCL15, which aids in avoiding off-target effects in human Panc-1 cells. As shown in Figure 3B and Figure S3, CCL15 protein expression was significantly decreased in Panc-1 cells stably expressing shCCL15 #1 or shCCL15 #2 as compared to the control cells. We next carried out a cell migration assay using these cells. We detected a significant reduction in cell migration ability in both Panc-1 shCCL15 #1 and Panc-1 shCCL15 #2 cells in comparison to the Panc-1 shScramble cells (Figure 3C). Furthermore, the same results were observed when recombinant human bFGF, a well-known wounding healing accelerator, was added to the migration assay as described above (Figure S4). To further strengthen our results from Panc-1 cells, we knocked down CCL9 in murine metastatic PDAC KP-2 cells using a lentiviral shRNA delivery system. As shown in Figure 3D, KP-2 cells infected by lentiviruses carrying shCCL9 #1 or shCCL9 #2 had decreased levels of CCL9 transcripts, down to 25–50% as compared to the control. Furthermore, these KP-2 cells with decreased CCL9 transcripts showed reduced cell migration abilities (Figure 3E). The blockade of CCL15 can be mediated through CCL15 gene knockdown or the neutralization of CCL15 via a CCL15-neutralizing antibody (NAb). We then tested Panc-1 cell migration abilities in the presence of a CCL15 NAb or a control isotype antibody. As shown in Figure 3F, neutralization of CCL15 in Panc-1 cells significantly impeded their migration ability. Furthermore, treating BxPC-3 cells with exogenously added recombinant CCL15 accelerated BxPC-3 mobility (Figure 3G).

**Figure 4:**
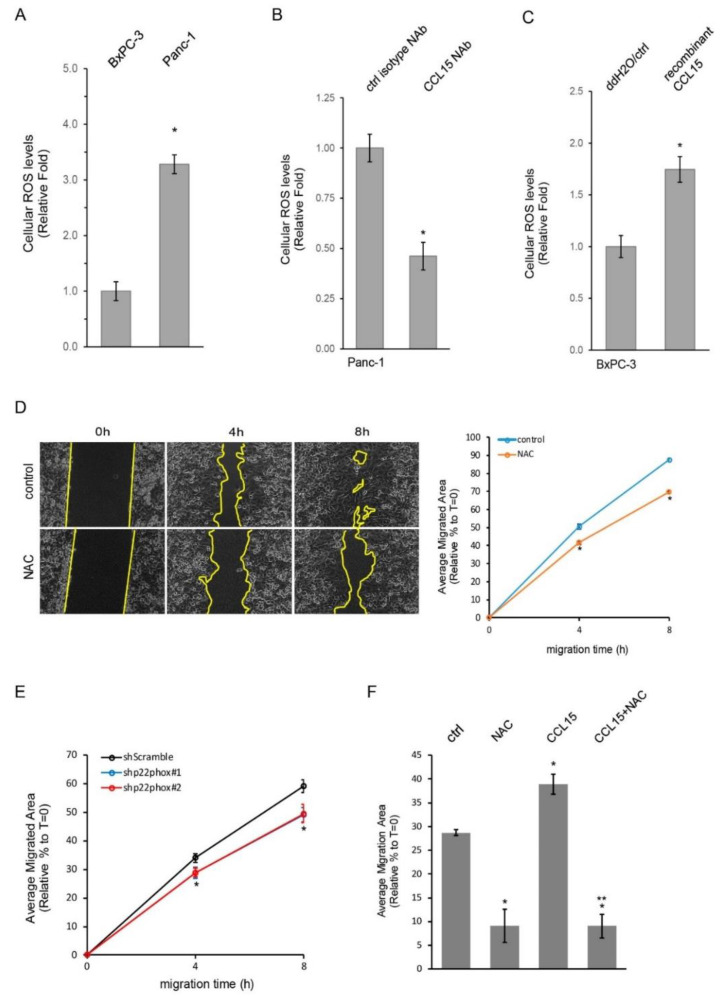
Depletion of reactive oxygen species attenuated CCL15-induced PDAC cell migration. (**A**) Cellular ROS levels of BxPC-3 and Panc-1 cells were detected as described in Section 2 and graphed. *: *p* < 0.05 as compared to BxPC-3. (**B**) Panc-1 cells were treated with an isotype control NAb or a CCL15 NAb (5 µg/mL) and subjected to detection of cellular ROS levels. *: *p* < 0.05 as compared to isotype NAb treatment. (**C**) BxPC-3 cells were treated with either control (ddH_2_O) or recombinant CCL15 (100 ng/mL) for 24 h followed by cellular ROS measurement. *: *p* < 0.05 as compared to control. (**D**) Panc-1 cells were seeded in culture-inserts as described in Section 2. After removal of culture-inserts, 5 mM NAC was added to the media, and Panc-1 cell migrated into the existing gap areas (yellow lines indicate the cell migration front lines) at the indicated time points and documented. The migrated areas were measured using Image J and calculated as a percentage relative to the areas at t = 0 in each condition. *: *p* < 0.05 as compared to control at the same migration time point. (**E**) Similar to (**D**), Panc-1 cells stably expressing shScramble, shp22phox #1, or shp22phox #2 were assessed for their cell migration ability at the indicated time points. *: *p* < 0.05 as compared to shScramble at the same migration time point. (**F**) BxPC-3 cells treated with 50 ng/mL recombinant CCL15 in the presence or absence of 5 mM NAC were examined for their cell migration abilities. *: *p* < 0.05 as compared to control. **: *p* < 0.05 as compared to recombinant CCL15.

As shown in Figure 4A, the levels of cellular ROS in Panc-1 cells were higher than those in BxPC-3 cells, which is positively associated with CCL15 expression as well as cell migration ability in these cells. Given that CCL15 is an inflammatory cytokine that associates with reactive oxygen species (ROS), we next tested whether the cellular ROS levels of Panc-1 and/or BxPC-3 cells are regulated by cytokine CCL15. When Panc-1 cells were treated with a specific CCL15 NAb, it reduced cellular ROS levels by 50% as compared to those treated with a control isotype NAb (Figure 4B). In contrast, the treatment of recombinant CCL15 significantly enhanced ROS production in BxPC-3 cells (Figure 4C). These results suggested a correlation among CCL15, ROS, and PDAC cell migration; therefore, we next examined whether ROS were involved in the cell motility of PDAC. When treating Panc-1 cells with the general ROS scavenger N-acetyl-L-cysteine (NAC), the migration speed of Panc-1 cells filling vacant areas was diminished (Figure 4D). The reduced cell migration ability of Panc-1 under NAC treatment was not due to the decreased cell proliferation of Panc-1 (Figure S5). Cellular ROS can be generated from many organelles such as mitochondria, endoplasmic reticulum, etc., and through cellular oxidative processes, including the activity of NADPH oxidases (NOXs) [26,27]. To determine whether NOXs were associated with PDAC cell motility, we knocked down p22phox, a critical component of NOXs [28,29] in Panc-1 cells, and examined the cell migration abilities of Panc-1 cells stably expressing shp22phox as well as Panc-1 shScramble control cells. As shown in Figure 4E and Figure S6, the knockdown of p22phox diminished Panc-1 cell mobility. To further dissect the relationship between ROS and CCL15-mediated cell migration, we treated BxPC-3 cells with recombinant CCL15, NAC, or both. As expected, treating BxPC-3 cells with exogenously added recombinant CCL15 accelerated their cell migratory ability, whereas NAC dramatically reduced it (Figure 4F). In addition, a dual treatment of NAC and recombinant CCL15 in BxPC-3 cells had the same effect as that of NAC treatment alone, suggesting that ROS are required for CCL15-mediated PDAC cell migration. Altogether, these data suggested that CCL15 promoted the cell mobility of PDAC through the upregulation of ROS.

### 3.4. CCL15 Promoted PDAC Cell Migration and Invasion and Was Mediated through Oncogenic Kras

**Figure 5:**
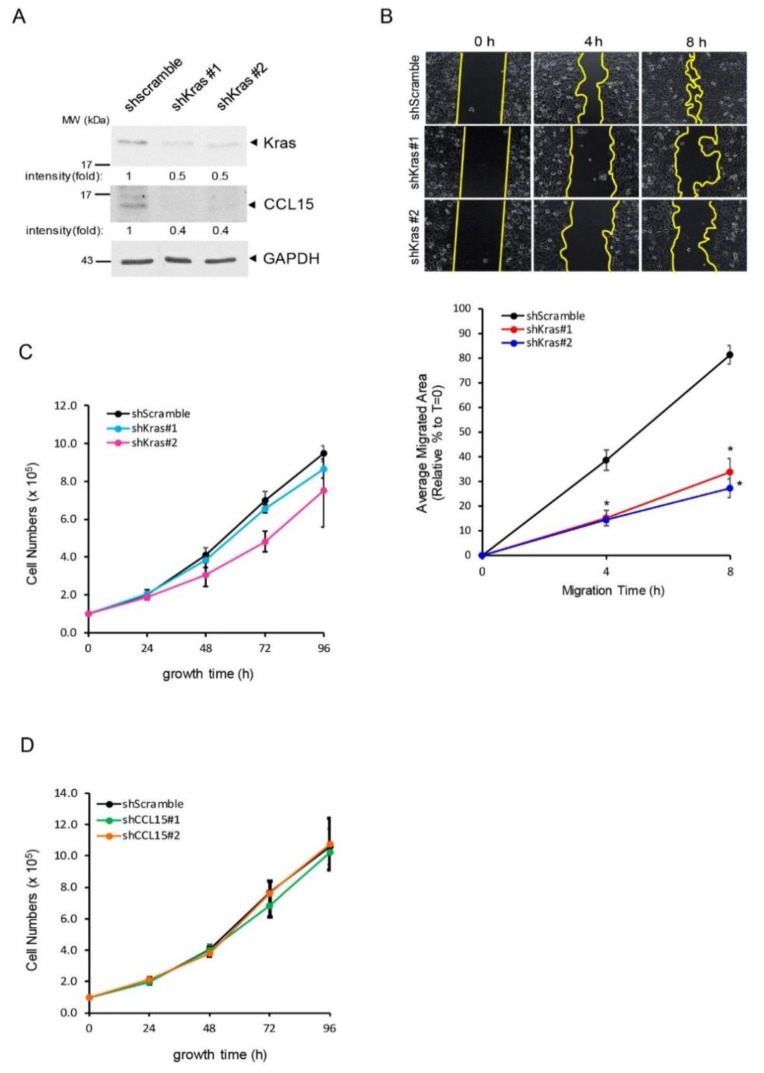
Oncogenic Kras-mediated CCL15 modulated cell migration of PDAC rather than PDAC cell growth. (**A**) Panc-1 cells stably expressing shScramble, shKras #1, or shKras #2 were examined for the expression levels of Kras and CCL15 using immunoblots. The expression of GAPDH was used as a loading control. Quantification result from 3 independent experiments is shown in Figure S7. (**B**) Panc-1 cells stably expressing shScramble, shKras #1, or shKras #2 were evaluated for their cell migration ability at the indicated time points. *: *p* < 0.05 as compared to shScramble at the same migration time point. (**C**) Panc-1 cells stably expressing shScramble, shKras #1, or shKras #2 were assessed for their cell growth over a 96 h time period. (**D**) Similar to (**C**), Panc-1 cells stably expressing shScramble, shCCL15 #1, or shCCL15 #2 were subjected to a cell proliferation assay. The cell numbers were counted over a 96 h time period. Original blots see Supplementary File S1.

Our results imply that oncogenic Kras mutation has the ability to regulate PDAC cellular migration by utilizing the CCL15/ROS axis. To further investigate this possibility, we knocked down Kras^G12D^ in highly migratory Panc-1 cells using lentiviral-delivered shRNA sequences that specifically target Kras and then assessed CCL15 expression as well as the cell migration ability in these cells (Figure 5A,B; Figure S7). As shown in Figure 5A, when 50% of Kras was knocked down, a simultaneous decrease in CCL15 expression in Panc-1cells was observed, suggesting that CCL15 is a downstream target of oncogenic Kras^G12D^. In addition, the knockdown of Kras^G12D^ significantly slowed the cell migration of Panc-1 by approximately 70% as compared to the control Panc-1 cells (Figure 5B), while similar observations were witnessed for CCL15 knockdown (Figure 3C). To assess whether Kras^G12D^ or CCL15 participates in PDAC cell growth, we also measured cell proliferation in the cells of Panc-1 of shscramble, shKras, and shCCL15. Neither the knockdown of Kras nor that of CCL15 affected Panc-1 cell growth (Figure 5C,D).

**Figure 6:**
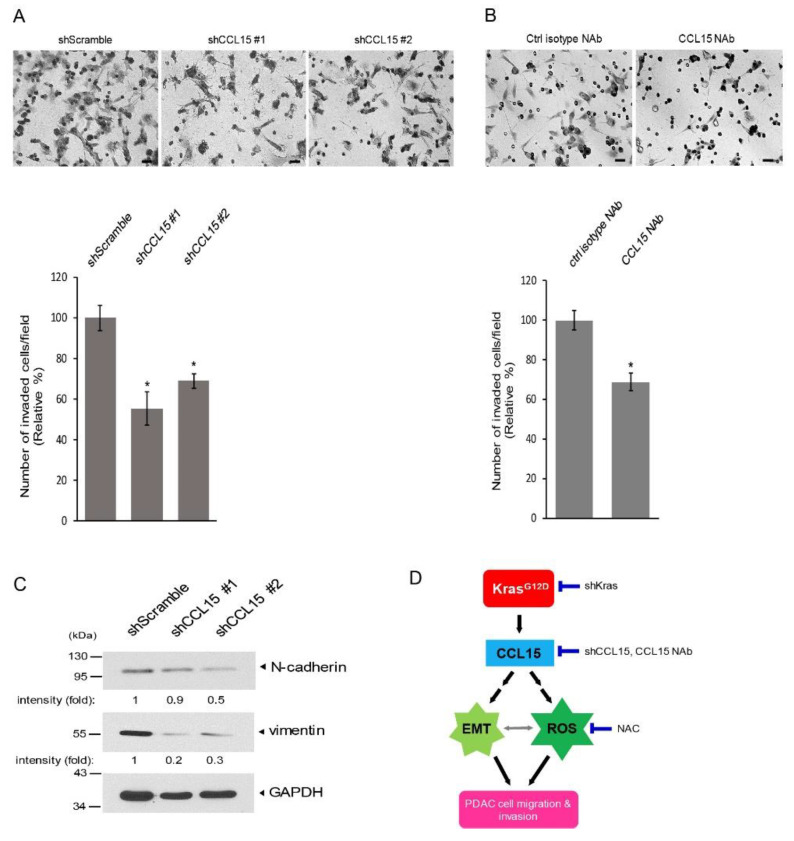
CCL15 accelerated PDAC cell invasion and regulated epithelial mesenchymal transition. (**A**) Human PDAC panc-1 cells stably expressing shScramble, shCCL15 #1, or shCCL15 #2 were subjected to a cell invasion assay as described in Section 2. The numbers of invaded Panc-1 cells in each condition were quantified by five randomly selected fields. The pictures shown are representative of three independent experiments. Scale bar: 50 µm. *: *p* < 0.05 as compared to shScramble. (**B**) Human PDAC panc-1 cells were subjected to a cell invasion assay in the presence of either a control isotype neutralizing antibody (NAb) or a CCL15 NAb. The numbers of invaded Panc-1 cells in each condition were quantified by five randomly selected fields. The pictures shown are representative of three independent experiments. Scale bar: 50 µm. *: *p* < 0.05 as compared to control NAb. (**C**) Expression levels of N-cadherin and vimentin in human PDAC Panc-1 cells that stably express shScramble, shCCL15 #1, or shCCL15 #2 were assessed by immunoblots. GAPDH levels were used as a control. Quantification result from 3 independent experiments is shown in Figure S8. (**D**) Schematic diagram to summarize findings. Original blots see Supplementary File S1.

Cancer metastasis requires cancer cells to invade the surrounding extracellular matrix and migrate out of the primary cancer site as the initial steps to reaching other organs. Prior to their invasion, cancer cells must detach from the epithelium while becoming more mobile via the epithelial mesenchymal transition (EMT) process. To test whether CCL15 can also regulate PDAC cell invasion, we evaluated the invasion ability of Panc-1 cells that stably expressed shCCL15 on matrigel. As shown in Figure 6A, decreased numbers of Panc-1 cells that had reduced levels of CCL15 expression (Figure 3B) invaded through a matrigel-coated membrane in comparison to the control cells, Panc-1 shScramble. Similarly, the neutralization of CCL15 from Panc-1 cells using a CCL15 NAb significantly suppressed their invasion into the matrigel (Figure 6B). To test the possibility of EMT regulation via CCL15, we utilized well-known EMT markers, including N-cadherin and vimentin, both of which are expressed by mensenchymal cells with high migratory abilities. As shown in Figure 6C and Figure S8, the knockdown of CCL15 reduced the expression levels of N-cadherin and vimentin. The same result was also observed in murine PDAC KP-2 cells when CCL9, a murine orthologue of CCL15, was knocked down (Figure S9), thus suggesting that this cytokine plays a critical role in the EMT process. Altogether, these data suggested that CCL15 positively regulates EMT to promote the cell invading ability of PDAC.

## 4. Discussion

Inflammation that causes elevated levels of ROS is pivotal to cancer initiation, development, and metastasis. In general, it is believed that ROS contribute to tumor progression through increased genomic instability [30]. It has been shown that oncogenes such as HRasV12, Kras, and c-Myc increase ROS levels in cancer cells [31]. During pancreatic cancer initiation, oncogenic Kras^G12D^ transdifferentiates acinar cells to a proliferating duct-like progenitor phenotype that later develops into pancreatic precancerous lesions through mitochondrial ROS [32]. Besides oncogenes, tumor suppressor genes such as p53, p21, and p16 also regulate ROS in non-cancerous cells by rendering them adapted to remodeled redox balance, which includes the upregulation of anti-oxidative as well as pro-apoptotic genes [33,34,35,36,37]. In addition, it has also been reported that tumor suppressor genes commonly lost in cancer metastasis modulate cell mobility and invasion. For example, the introduction of BTG2, a tumor suppressor gene lost in metastatic lung and prostate cancers, repressed cell migration and cancer invasion by the inhibition of mitochondrial ROS as well as Src activity [38].

Redox regulation, especially through ROS production, is well known to modulate cellular migration and adhesion process [39]. Our data showed that a general ROS scavenger, N-Acetyl-L-Cysteine (NAC), dramatically reduced the cell migration of Panc-1 cells that expressed high levels of CCL15 (Figure 4D). Furthermore, the decrease in the cell migratory abilities of Panc-1 in the presence of a depleting ROS was not due to reduced cell proliferation of Panc-1 (Figure S5). In fact, we actually detected a higher DNA content of Panc-1 treated with NAC in comparison to the control cells; this result further supports the conclusion that ROS positively modulates Panc-1 cell migration. Interestingly, the depletion of ROS through the knockdown of p22phox, a key component of NOXs controlling cellular ROS levels [28,40], also slowed down Panc-1 cell migration (Figure 4E), suggesting that NOXs are one of the resources used by ROS to mediate PDAC cell migration. Not surprisingly, it has been demonstrated that the knockdown of Nox4 blocked TGF-β-mediated Panc-1 epithelial-mesenchymal transition (EMT), thus decreasing cell migration [41]. Furthermore, the activation of Kras and inactivation of p16 in immortalized human pancreatic epithelial cells led to the upregulated expression of Nox4 and p22phox and increased the activity of NOX [42]. In addition, the knockdown of either Nox4 or p22phox reduced glycolysis in human PDAC cell lines and PDAC cell growth, both in vitro and in xenograft mice.

So far, various mechanisms demonstrating how oncogenic Kras mutations regulate PDAC cell migration and invasion have been reported. These include its regulation through NF-κB, p53, Src, RKIP and even post-translational modifications of mutant Kras such as sumoylation [43,44,45,46,47]. In this current study, our results demonstrated that a dual treatment of NAC and recombinant CCL15 in BxPC-3 cells completely abolished the exogenously added recombinant CCL15-induced cell migration of BxPC-3 cells (Figure 4F). This suggests that ROS function as the downstream target of CCL15 to mediate PDAC cell migration. Furthermore, the knockdown of Kras in Panc-1 cells diminished CCL15 protein expression levels (Figure 5A). Both CCR1 and CCR3, receptors for CCL15, were expressed in PDAC cells (Figure 1B,C and Figure 2C,E). These data imply that oncogenic Kras upregulates CCL15 in PDAC cells, allowing CCL15 to bind to its receptors on the PDAC cells, which leads to the elevated production of ROS and subsequently increased PDAC cell migration and invasion. This autocrine signaling mechanism is commonly utilized by PDAC cells to maximize their own capacity for cell growth, metastasis, and drug resistance [48]. However, whether these previously reported mechanisms involved in NF-κB, p53, Src and/or RKIP are also associated with our newly discovered mechanism requires further investigation. Besides CCL15 which we reported here the newly identified function of expediting PDAC cell migration and invasion, other cytokines that have been reported to promote EMT of PDAC through an autocrine mechanism include IL-6, IL-8, TGF-β, CCL2, and CXCL12 [49,50,51,52,53,54,55,56,57].

## 5. Conclusions

In summary, we provided evidence that demonstrates a novel function of CCL15, namely mediating PDAC cell migration and invasion by increasing cellular ROS levels (Figure 6D). In addition, upregulated levels of CCL15 expression in PDAC cells were positively associated with oncogenic Kras mutants, and the knockdown of Kras abolished CCL15 expression, which suggests that oncogenic Kras mutations regulate CCL15 protein expression in PDAC cells. The expression of CCL15 and its receptors CCR1 and CCR3 was present in PDAC cells, in both cell lines and tissue samples. Altogether, our results revealed another mechanism that PDAC cells utilize to self-promote cancer dissemination through oncogenic KRas mutations and its downstream targets CCL15 and ROS in order to enhance cell migration and invasion.

## Data Availability

Data available is contained within the article and Supplementary Materials.

## Supplementary Materials

The following supporting information can be downloaded at: https://www.mdpi.com/article/10.3390/cancers14092153/s1, Figure S1: CCL15 gene amplification is only present in PDAC and may be prognostic factor for overall survival of PDAC, Figure S2: Increased expression of CCL9 in murine metastatic PDAC, Figure S3:Quantification of CCL15 knockdown efficiency in Panc-1 cells, Figure S4: Knockdown of CCL15 decreased Panc-1 cell migration in the presence of bFGF, Figure S5: Inhibition of ROS by NAC treatment increased cell proliferation of Panc-1, Figure S6: Levels of p22phox expression in Panc-1 cells that stably expressing shScramble and shp22phox, Figure S7: Quantification of levels of Kras and CCL15 in Panc-1 cells that stably expressing shScramble and shKras, Figure S8: Quantification of levels of N-cadherin and vimentin in Panc-1 cells, Figure S9: Knockdown of CCL9 in murine PDAC KP-2 cells reduced N-cadherin, Figure S10: Specificity of CCR1 and CCR3 antibody used in immunohistochemistry. File S1: Original blots.
