# Supplementary material for: Oncogenic Kras-Mediated Cytokine CCL15 Regulates Pancreatic Cancer Cell Migration and Invasion through ROS
Source: Cancers (Basel). 2022 Apr 26;14(9):2153. doi: 10.3390/cancers14092153 (PMC9104113; doi:10.3390/cancers14092153)
Supplement: Supplementary file 1 [file cancers-14-02153-s001.zip › cancers-1674348-supplementary figure.pdf]

Supplemental Figure S1

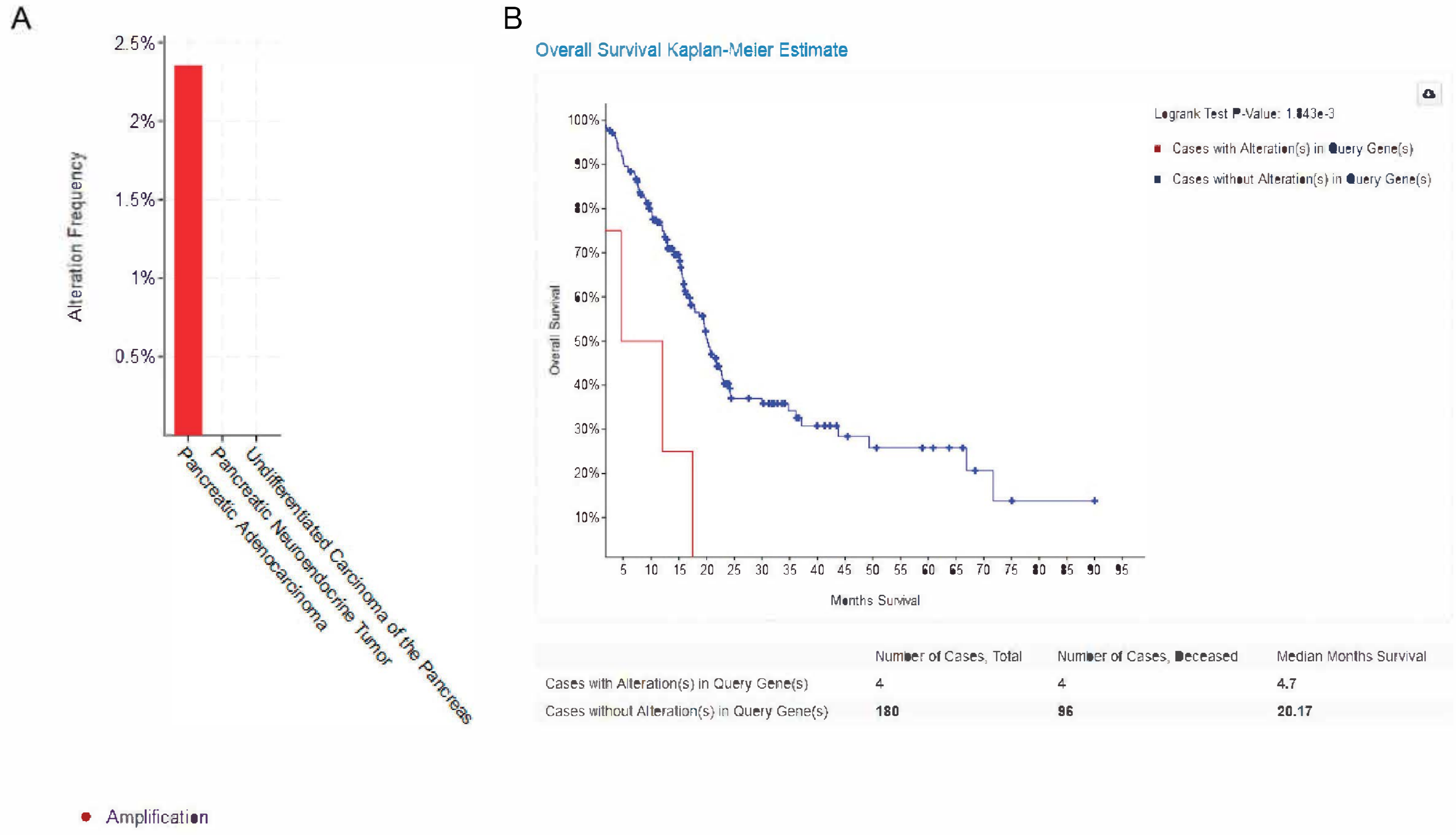

	Number of Cases, Total	Number of Cases, Deceased	Median Months Survival
Cases with Alteration(s) in Query Gene(s)	4	4	4.7
Cases without Alteration(s) in Query Gene(s)	180	96	20.17

Supplemental Figure S1. CCL15 gene amplification is only present in PDAC and may be a prognostic factor for overall survival of PDAC. (A) CCL15 gene amplification data from different types of pancreatic cancer including PDAC, pancreatic neuroendocrine tumors and undifferentiated carcinoma of the pancreas were obtained from the TCGA data (cBioPortal for Cancer Genomics). (B) The overall survival rate from 184 cases of PDAC patients according to their CCL15 gene amplification status was plotted. There were 180 cases without CCL15 gene amplification (blue) and 4 cases with CCL15 gene amplification (red). Without CCL15 amplification, the median months survival was 20.17 months; whereas 4.7 months for patients who had CCL15 amplification.This data was obtained from the existing TCGA data.

Supplemental Figure S2

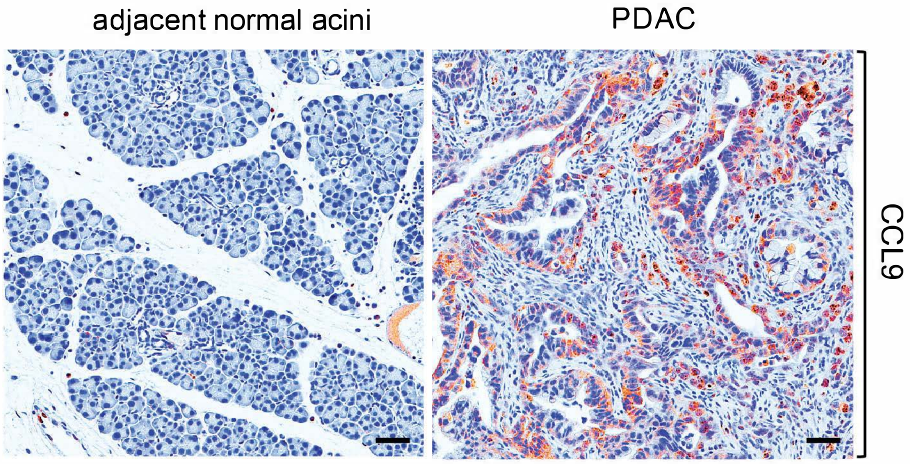

Supplemental Figure S2. Increased expression of CCL9 in murine metastatic PDAC. Pancreas tissue samples from KPC mice at age of 26 weeks that developed metastatic PDAC were immunostained with a CCL9 antibody. The adjacent normal pancreas area containing pancreatic acini was used as a control. Scale bar: 50 µm.

Supplemental Figure S3

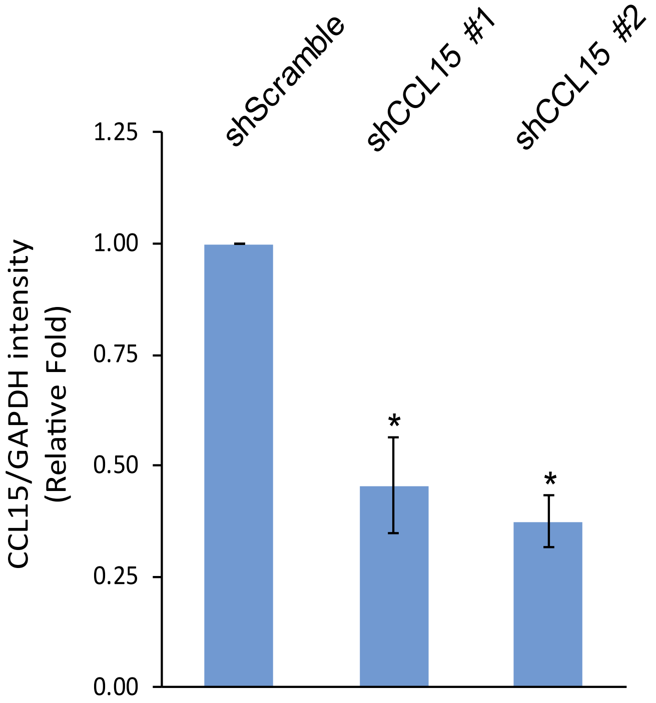

Supplemental Figure S3. Quantification of CCL15 knockdown efficiency in Panc-1 cells. Cell lysates of Panc-1 cells stably expressing shScramble, shCCL15#1 and shCCL15#2 were subjected to immunoblots of CCL15 and GAPDH as shown in Fig. 3B. Band intensity of CCL15 and GAPDH was quantified and graphed as in relative fold to shScramble. 4 independent experiments were graphed here and *: p<0.05 as compared to shScramble.

Supplemental Figure S4

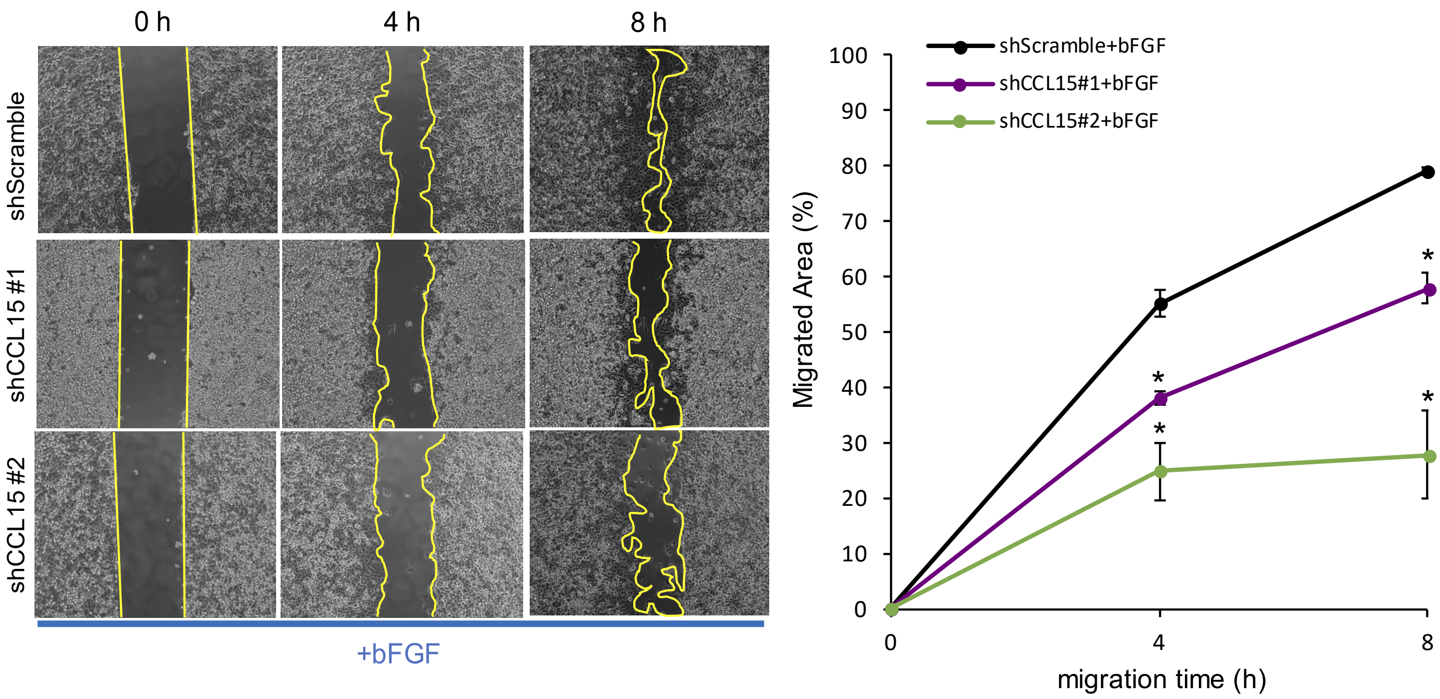

Supplemental Figure S4. Knockdown of CCL15 decreased Panc-1 cell migration in the presence of bFGF. As described in the Materials and Methods section, Panc-1 cells stably expressing shCCL15 were subjected to cell migration assay in the presence of 100 ng/mL recombinant human bFGF for the indicated time period. *: $p<0.05$ as compared to shScramble at the same migration time period.

Supplemental Figure S5

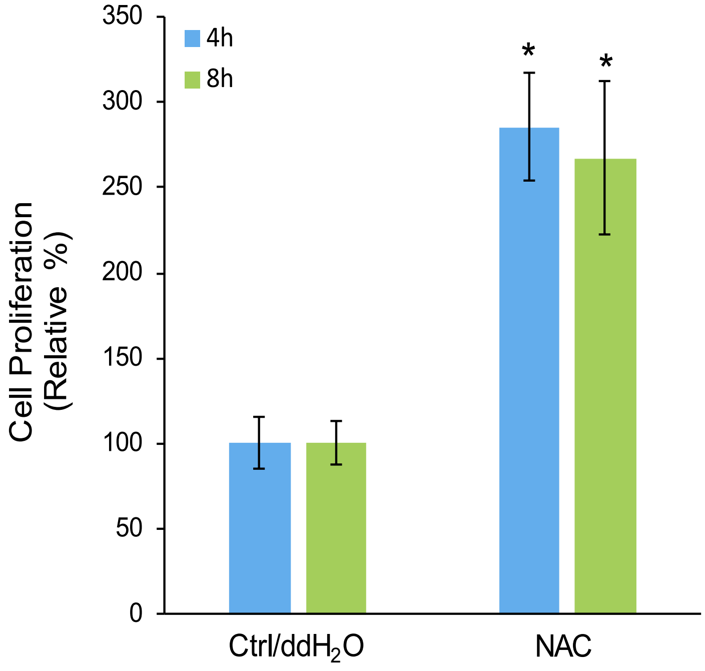

Supplemental Figure S5. Inhibition of ROS by NAC treatment increased cell proliferation of Panc-1. Cell proliferation of Panc-1 cells treated either with control or 5 mM NAC for the indicated time periods was evaluated by CyQuant assay which determines DNA content. In brief, 2000 cells per well were plated in a 96 well plate in complete media containing 10% FBS. Next day, cells were washed with PBS twice and the migration media (1% FBS) containing with either water or 5 mM NAC were added to the cells for 4 h and 8h. At the indicated time points, the cells were washed and lysed according to the manufactures' menu. The DNA content was measured using the CyQuant GR kit in a plate reader with Ex/Em 485/528. DNA content in each condition was graphed in a relative fold to control at each indicated time point. *: $p<0.05$ as compared to control.

Supplemental Figure S6

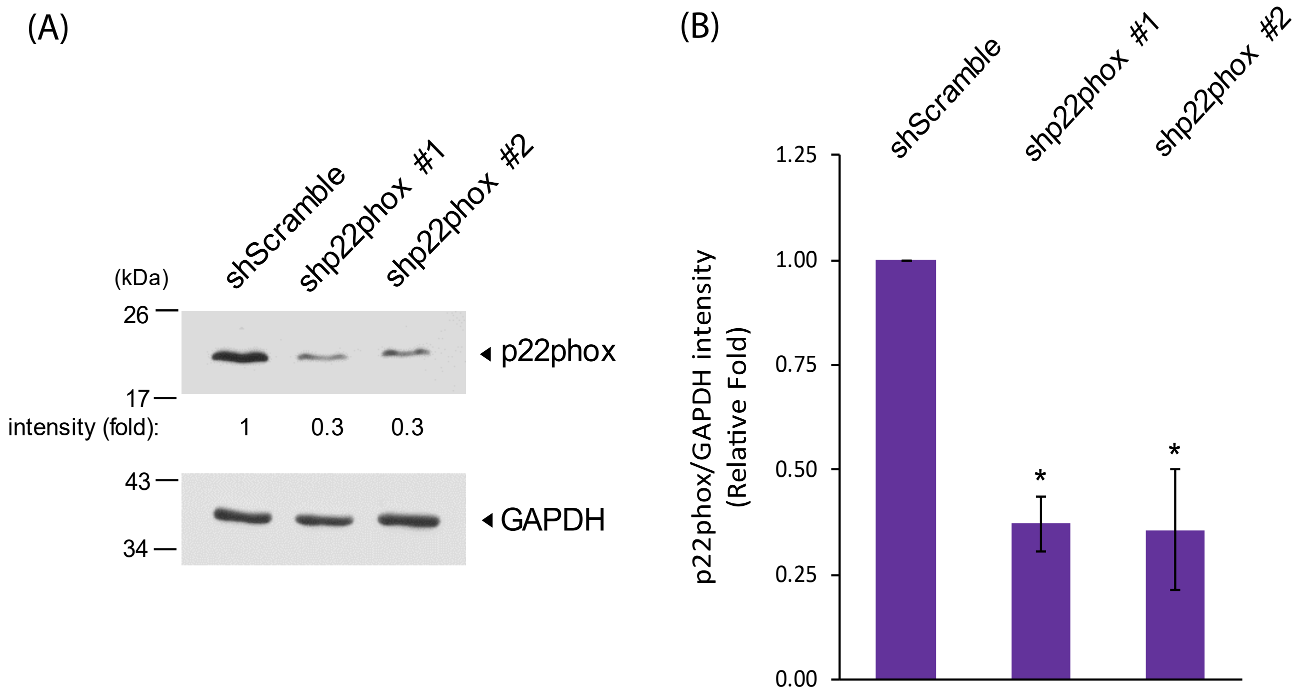

Supplemental Figure S6. Levels of p22phox expression in Panc-1 cells that stably expressing shScramble and shp22phox. (A) Cell lysates of Panc-1 cells stably expressing shScramble, shp22-phox #1 and shp22phox #2 were subjected to immunoblots of p22phox and GAPDH. (B) Band intensity of p22phox and GAPDH was quantified and graphed as in relative fold to shScramble. 4 independent experiments were graphed. *: $p<0.05$ as compared to shScramble.

Supplemental Figure S7

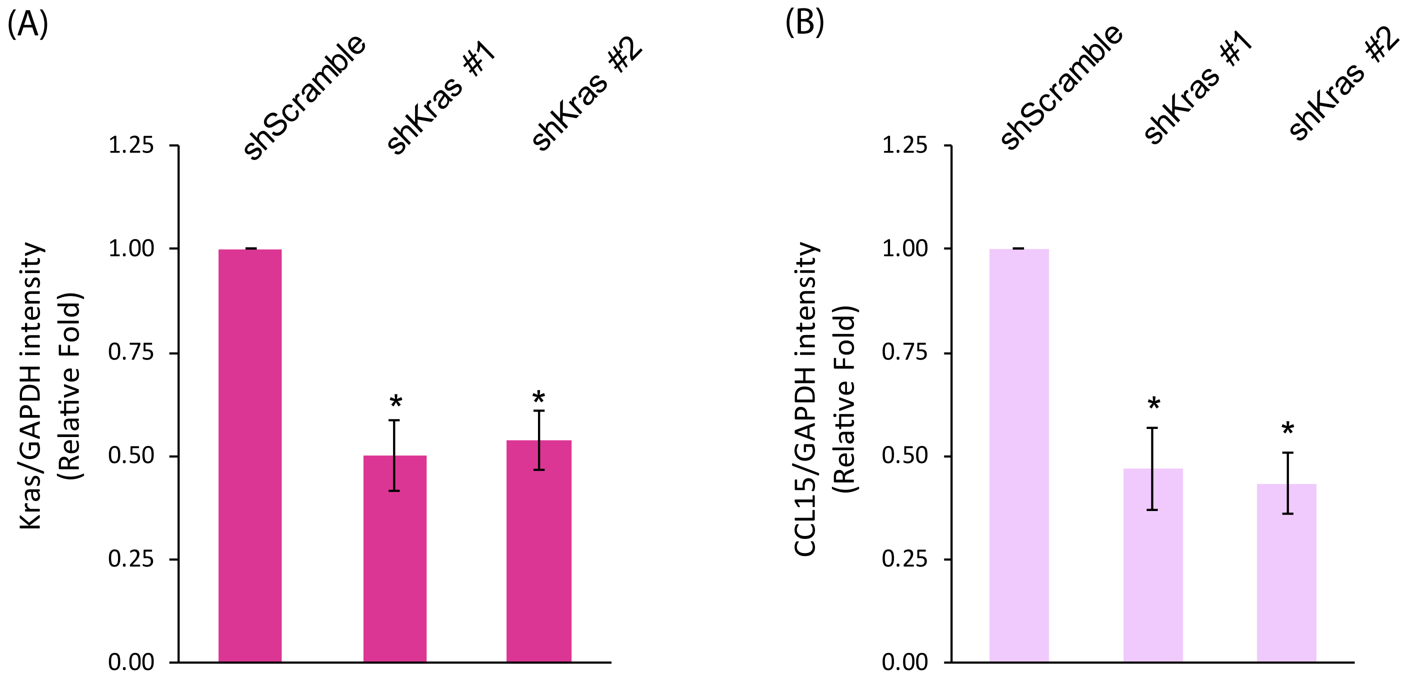

Supplemental Figure S7. Quantification of levels of Kras and CCL15 in Panc-1 cells that stably expressing shScramble and shKras. Cell lysates of Panc-1 cells stably expressing shScramble, shKras #1 and shKras #2 were subjected to immunoblots of Kras, CCL15 and GAPDH as shown in Fig. 5A. Band intensity of (A) Kras, (B) CCL15 and GAPDH was quantified and graphed as in relative fold to shScramble. At least 3 independent experiments were graphed here and *: $p<0.05$ as compared to shScramble.

Supplemental Figure S8

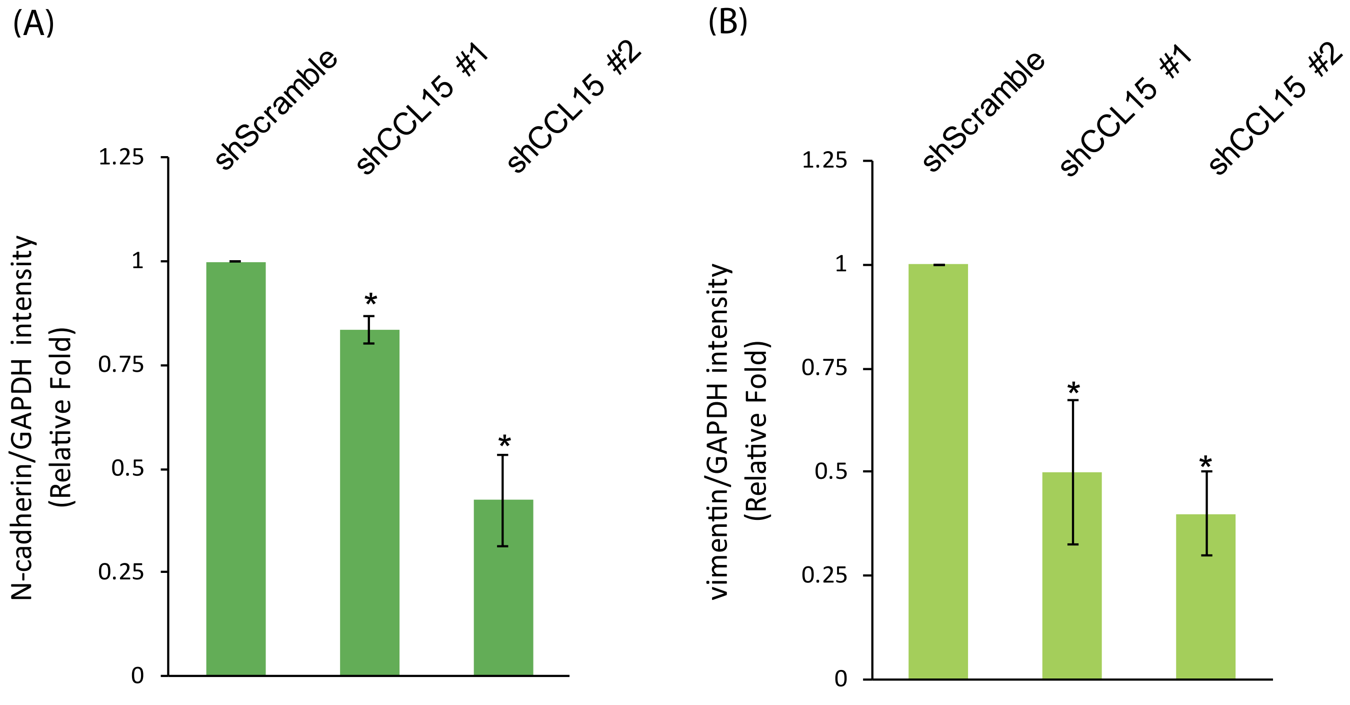

Supplemental Figure S8. Quantification of levels of N-cadherin and vimentin in Panc-1 cells that stably expressing shScramble and shCCL15. Cell lysates of Panc-1 cells stably expressing shScramble, shCCL15 #1 and shCCL15 #2 were subjected to immunoblots of N-cadherin, vimentin and GAPDH as shown in Fig. 6C. Band intensity of (A) N-cadherin, (B) vimentin and GAPDH was quantified and graphed as in relative fold to shScramble. 3 independent experiments were graphed here and *: $p<0.05$ as compared to shScramble.

Supplemental Figure S9

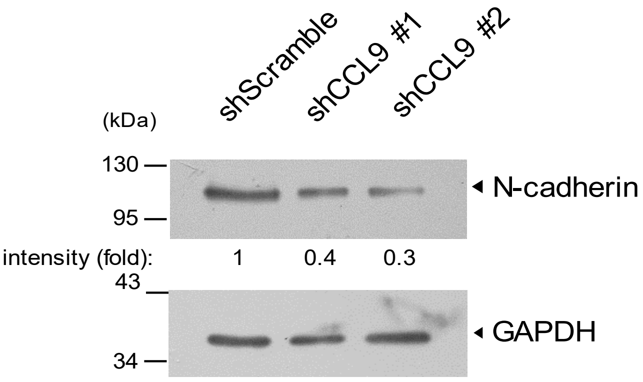

Supplemetal Figure S9. Knockdown of CCL9 in murine PDAC KP-2 cells reduced N-cadherin expression. Levels of N-cadherin, a marker for epithelial mesenchymal transition were examined muirne PDAC KP-2 cells stably expressing shScramble, shCCL9 #1 or shCCL9 #2 by immunoblots. GAPDH protein levels were used as a loading control. The intensity of N-cadherin of each lane was indicated as a relative fold to the ratio of N-cadherin/GAPDH of shScramble.

Supplemental Figure S10

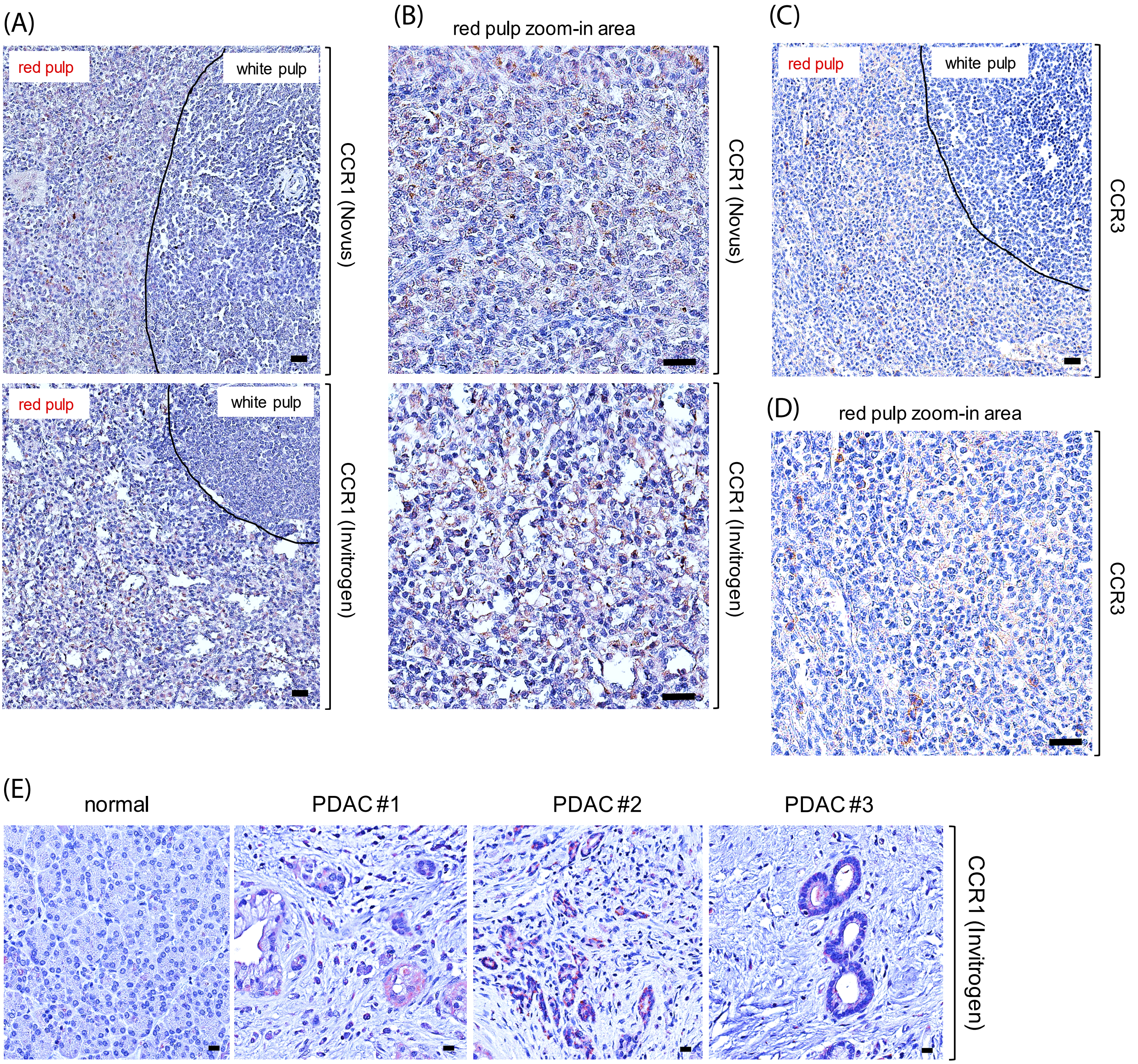

Supplemental Figure S10. Specificity of CCR1 and CCR3 antibody used in immunohistochemistry. (A-D) human spleen tissue samples were immunostained with CCR1 antibodies from Novus and Invitrogen and CCR3 antibody from Abcam. (A) Both CCR1 antibodies only stained the red pulp area but not white pulp area of the spleen. (B) Red-pulp zoom-in areas of the spleen that were positive for CCR1. (C) CCR3 antibody only stained the red pulp areas of the spleen instead of the white pulp areas. (D) Red-pulp zoom-in areas of the spleen that were CCR3-positive. Scale bar: 100 μm. (E) Another set of PDAC samples and the normal human pancreas stained with CCR1 antibody from Invitrogen. scale bar: 50 μm.
