# Supplementary material for: Oncogenic Kras-Mediated Cytokine CCL15 Regulates Pancreatic Cancer Cell Migration and Invasion through ROS
Source: Cancers (Basel). 2022 Apr 26;14(9):2153. doi: 10.3390/cancers14092153 (PMC9104113; doi:10.3390/cancers14092153)
Supplement: Supplementary file 1 [file cancers-14-02153-s001.zip › File S1/original blots-main text.pdf]

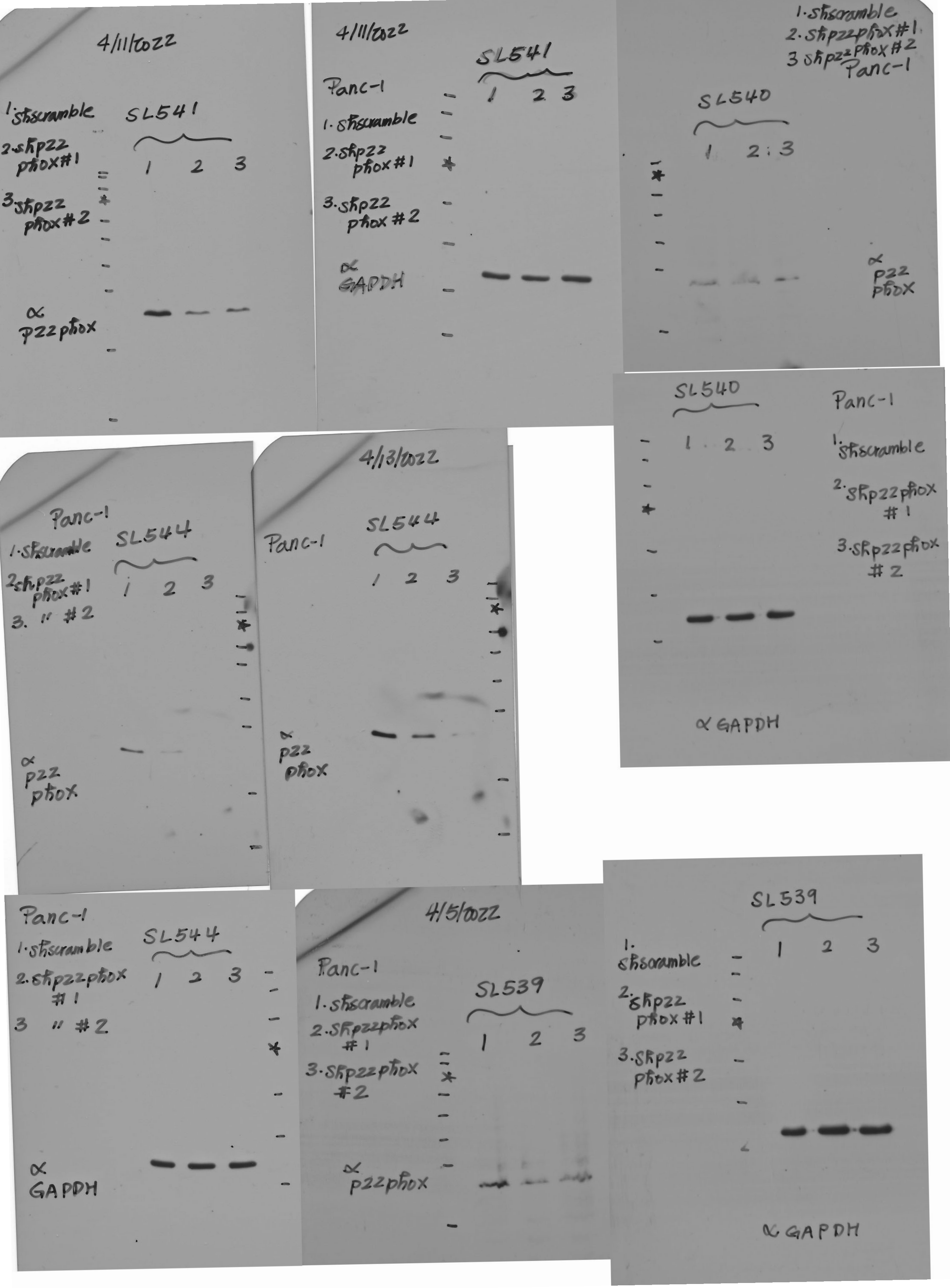
4/11/2022
1. shscramble
2. shp22 phox#1
3. shp22 phox#2
SL541
1 2 3
α p22phox
4/11/2022
Panc-1
1. shscramble
2. shp22 phox#1
3. shp22 phox#2
SL541
1 2 3
α GAPDH
1. shscramble
2. shp22phox#1
3 shp22phox#2
Panc-1
SL540
1 2 3
α p22 phox
SL540
Panc-1
1 2 3
1. shscramble
2. shp22phox #1
3. shp22phox #2
α GAPDH
Panc-1
1. shscramble
2 shp22 phox#1
3. " #2
SL544
1 2 3
α p22 phox
4/13/2022
Panc-1
SL544
1 2 3
α p22 phox
Panc-1
1. shscramble
2. shp22phox #1
3 " #2
SL544
1 2 3
α GAPDH
4/5/2022
Panc-1
1. shscramble
2. shp22phox #1
3. shp22phox #2
SL539
1 2 3
α p22phox
SL539
1. shscramble
2. shp22 phox#1
3. shp22 phox#2
1 2 3
α GAPDH

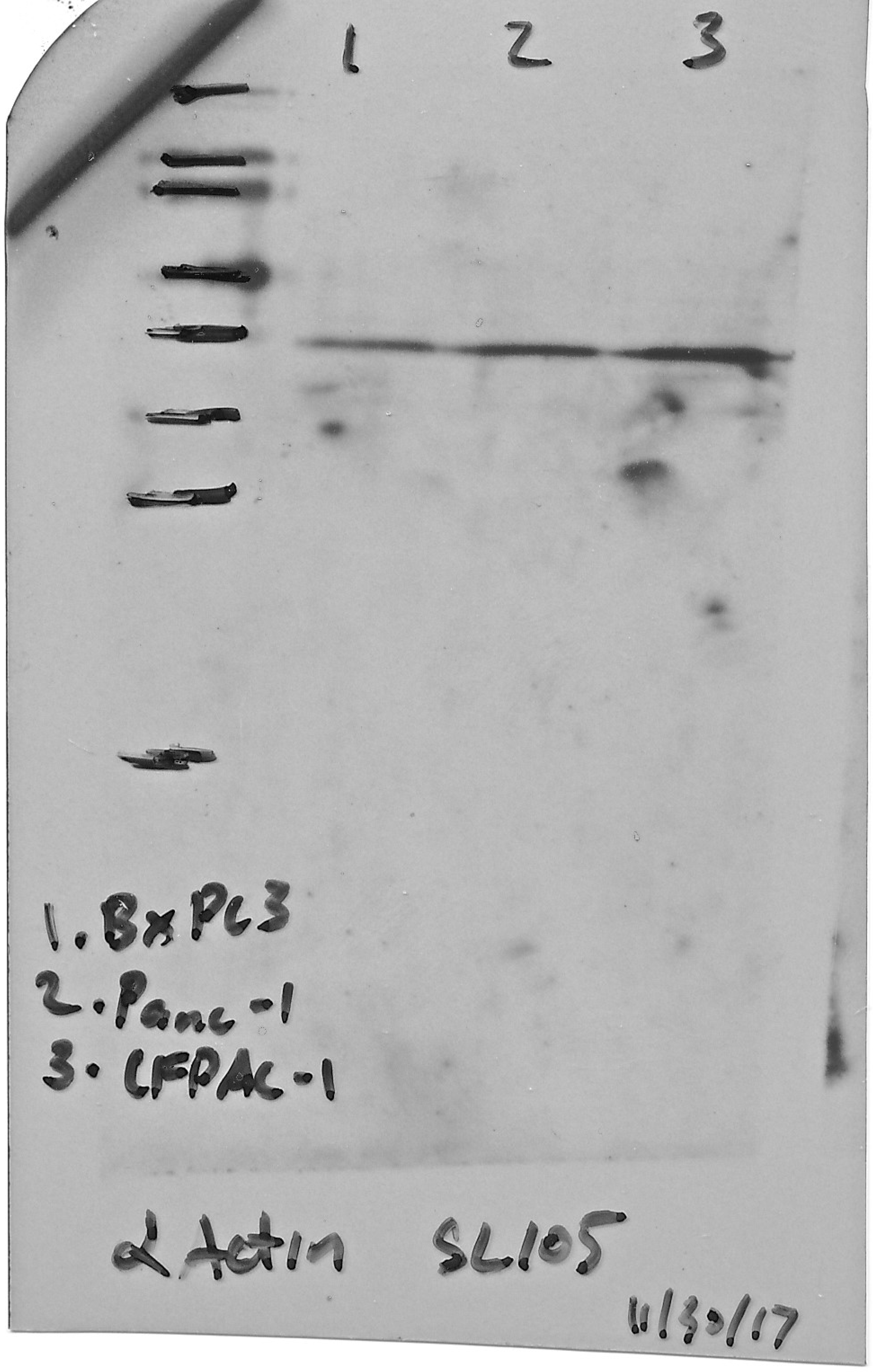

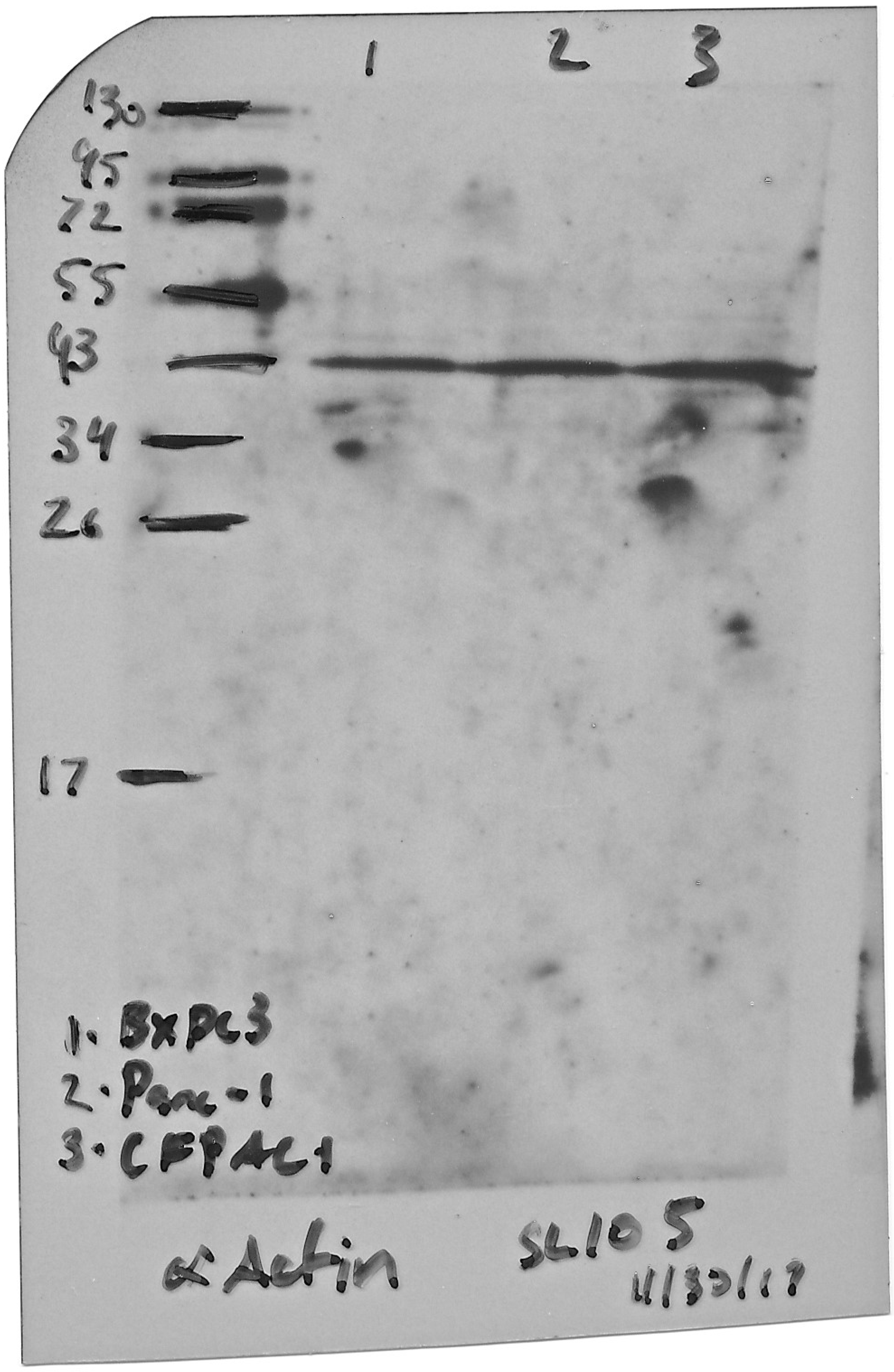

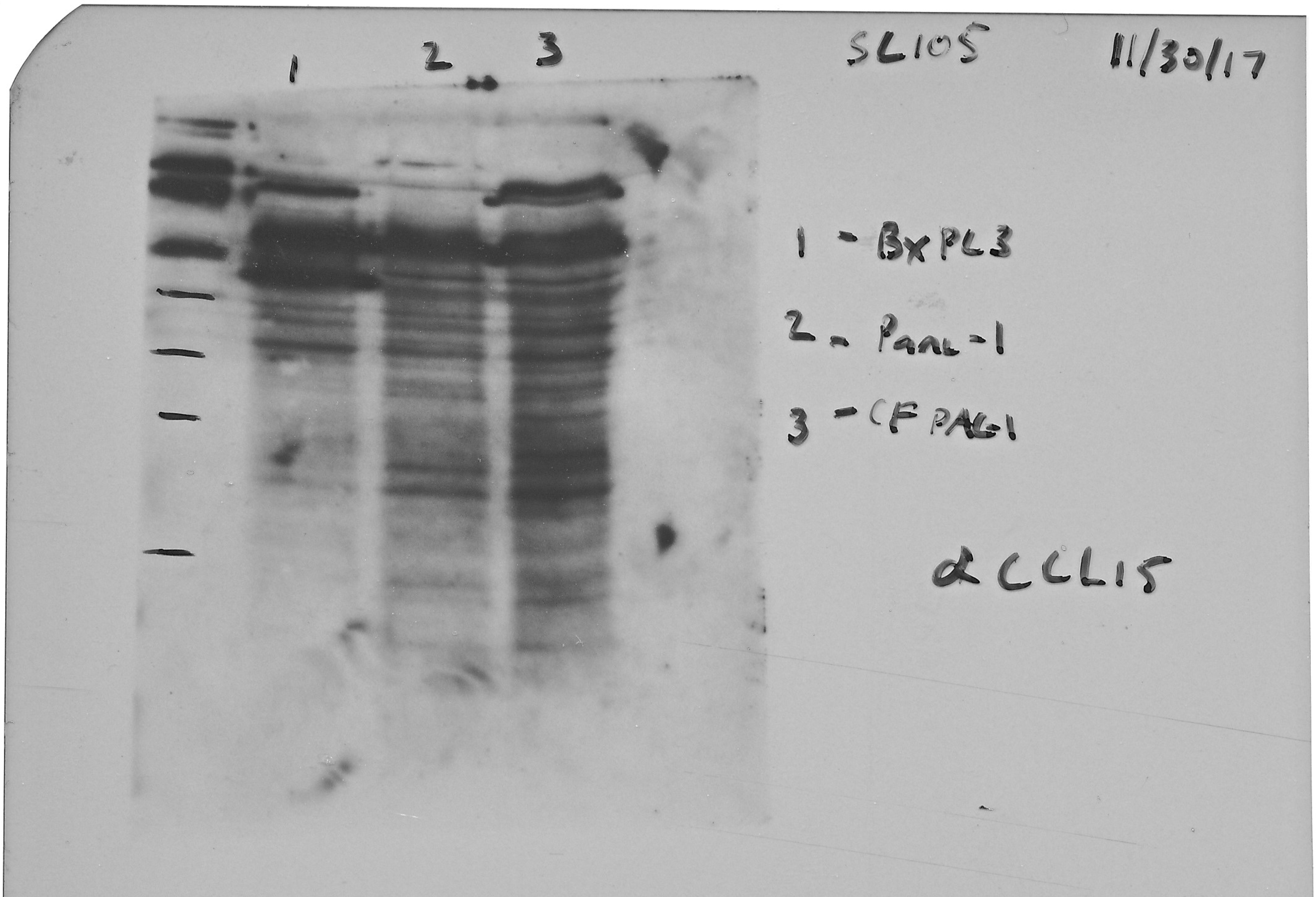

Figure 2B original blots

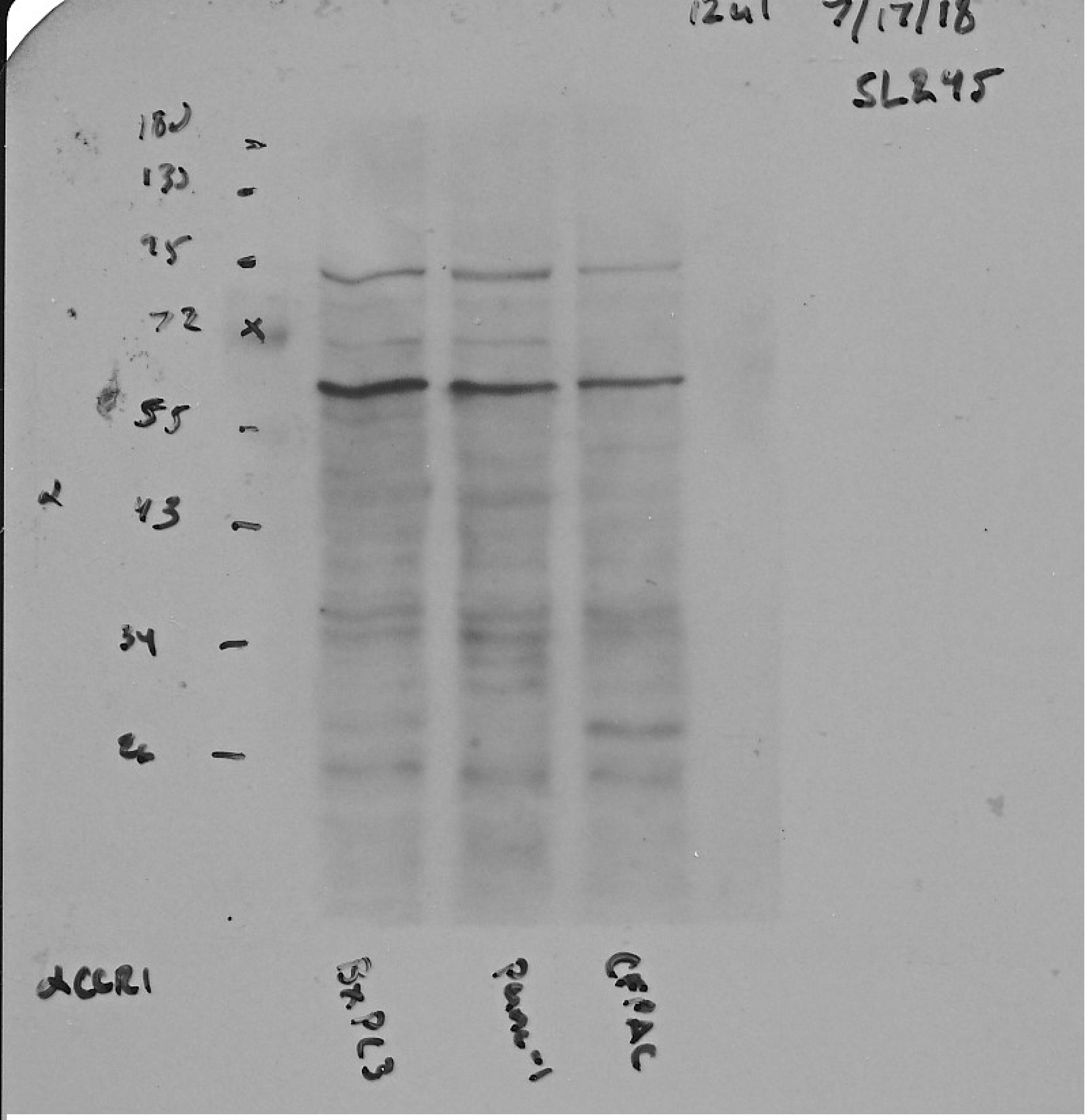

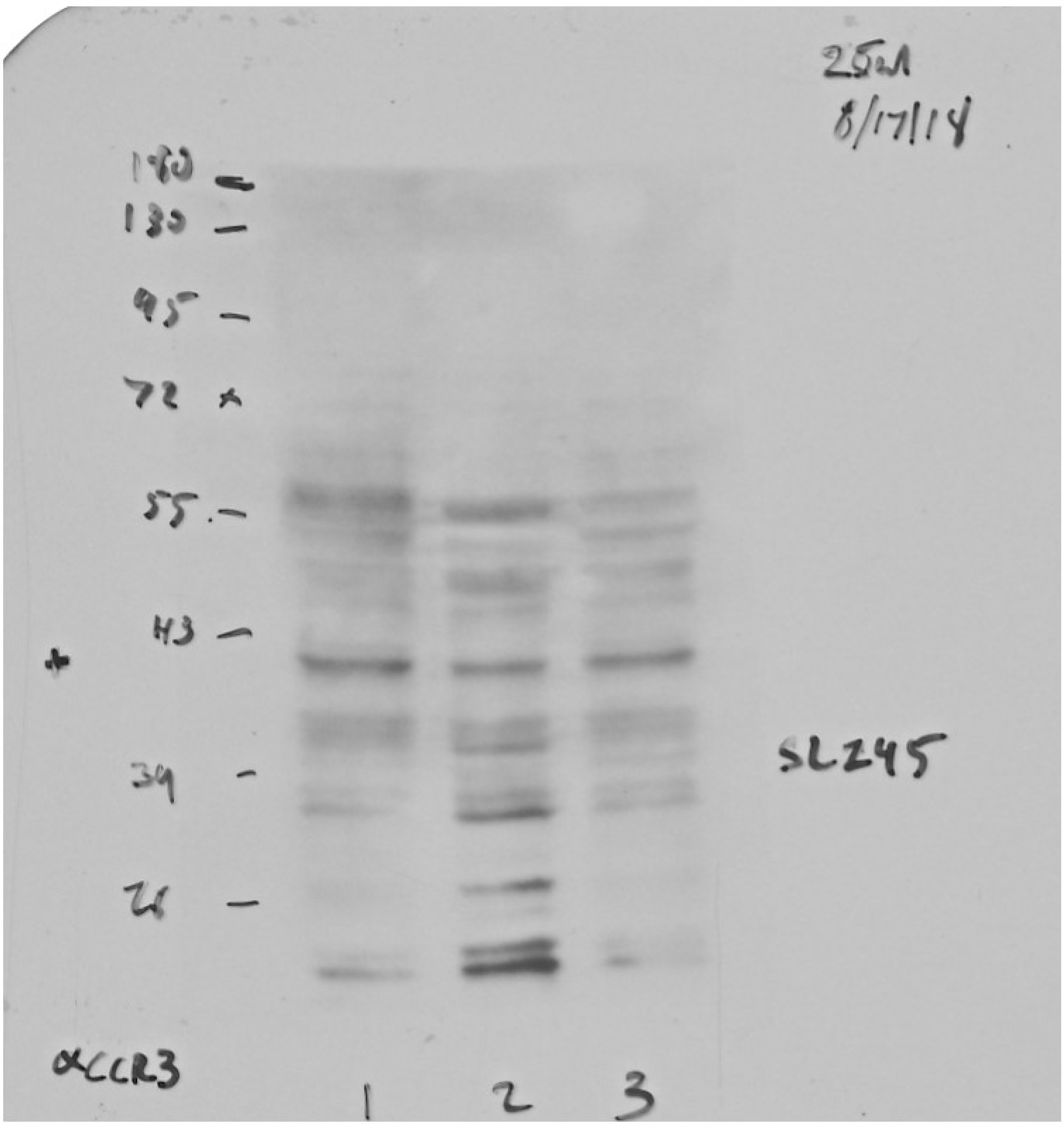

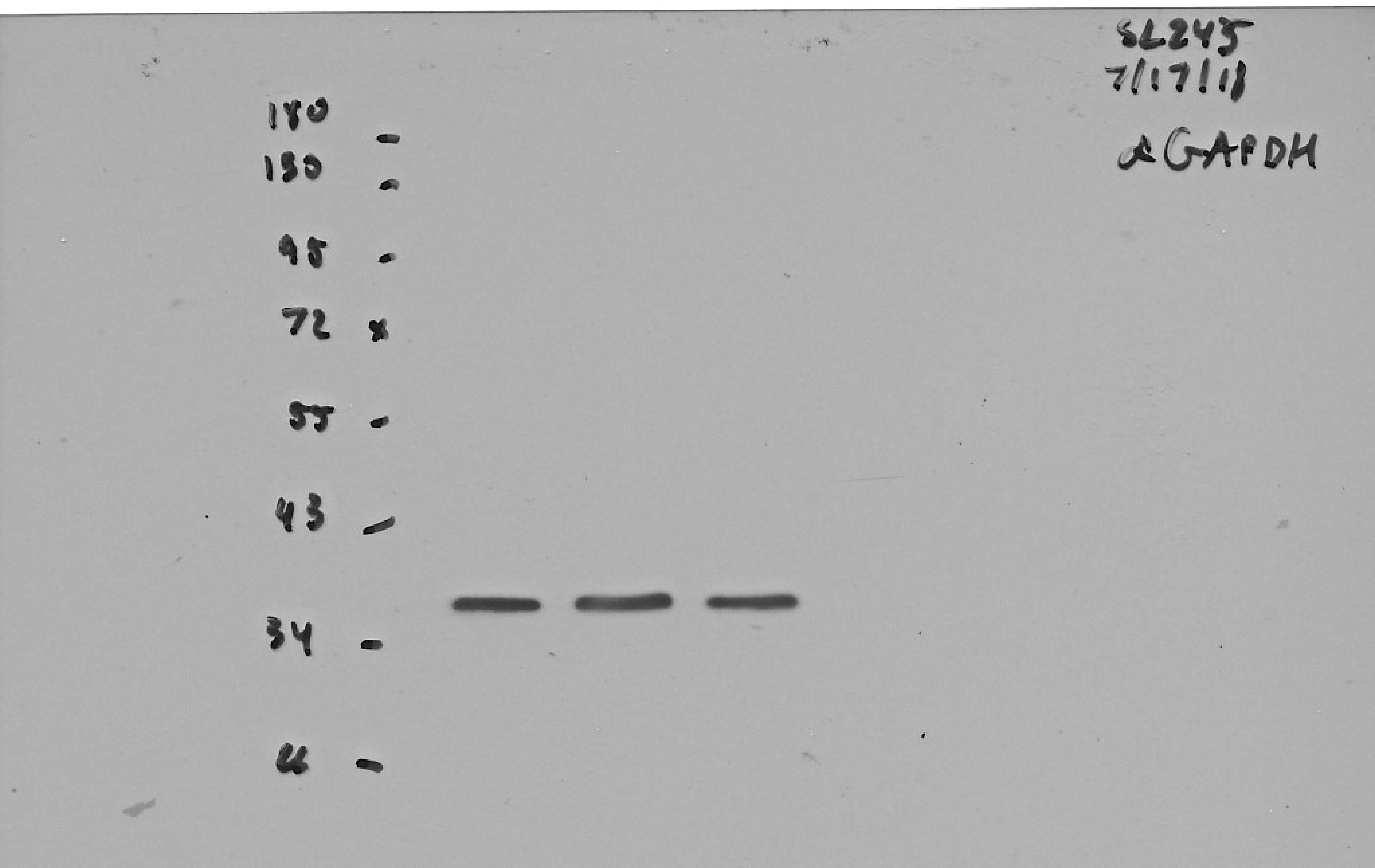

Figure 2C original blots

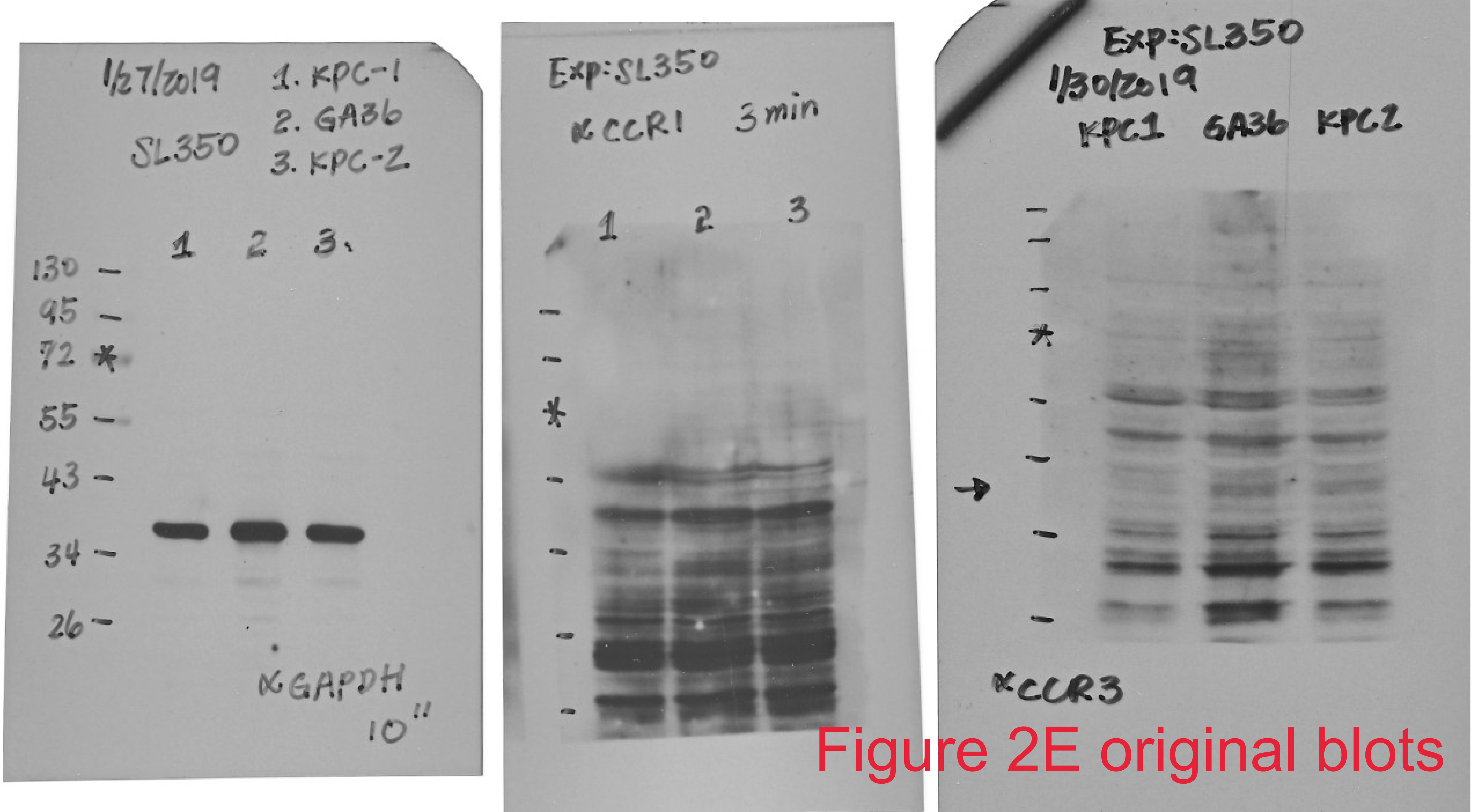

Figure 2E original blots

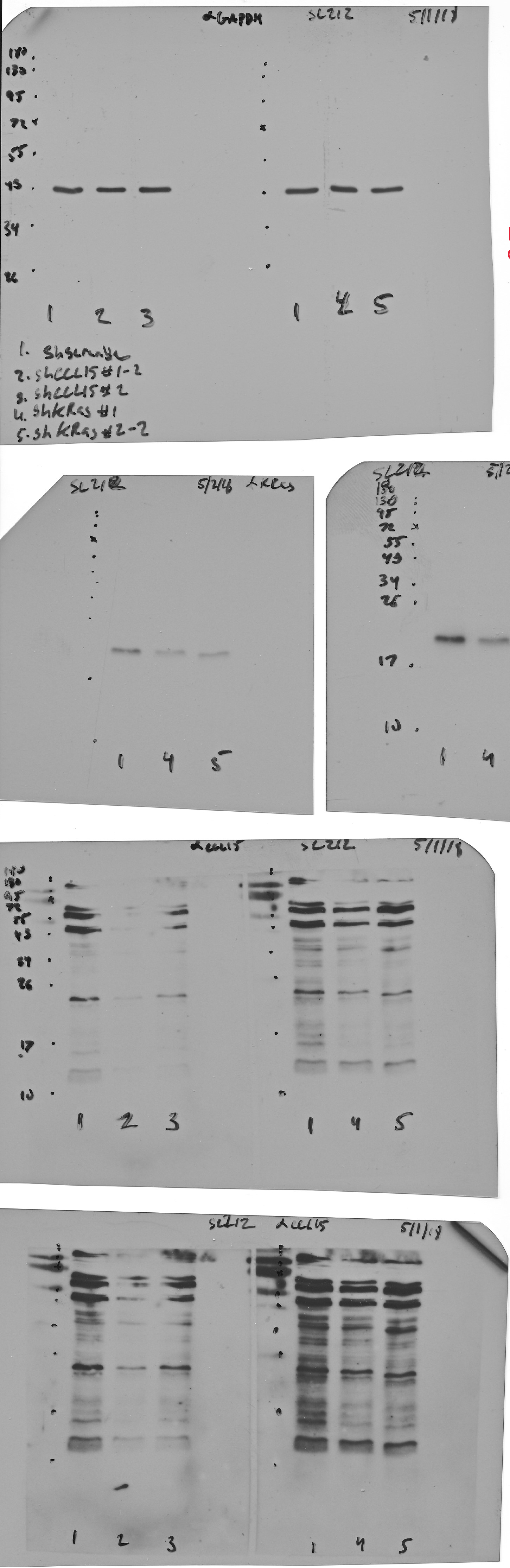

Figures 3B & 5A original blots

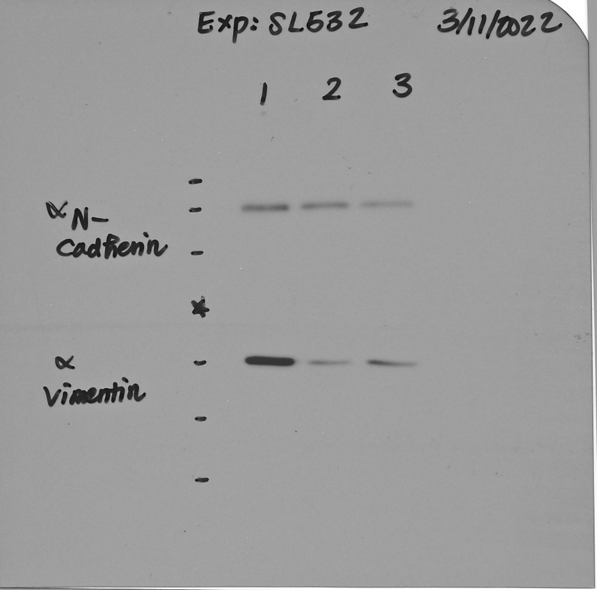

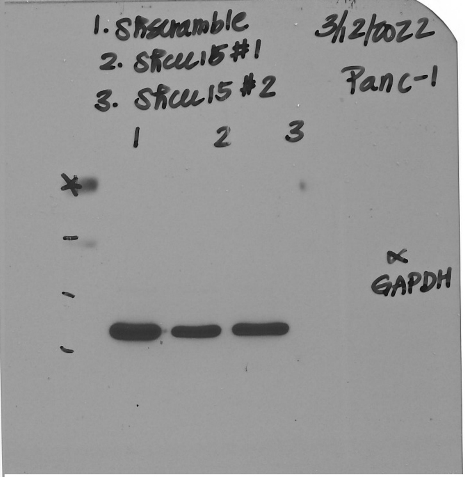

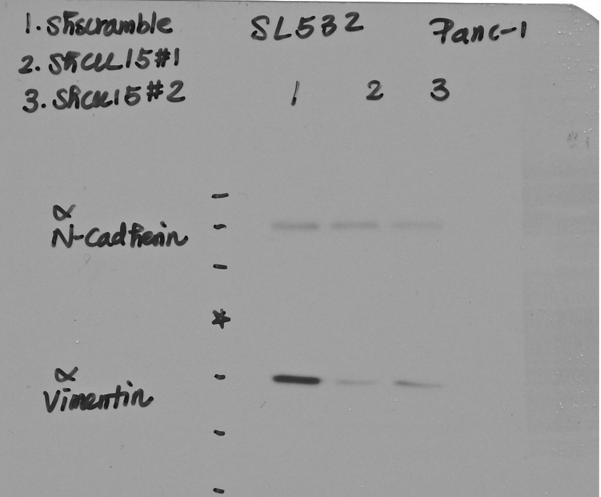

Fig 6C original blots
