# Supplementary material for: Oncogenic Kras-Mediated Cytokine CCL15 Regulates Pancreatic Cancer Cell Migration and Invasion through ROS
Source: Cancers (Basel). 2022 Apr 26;14(9):2153. doi: 10.3390/cancers14092153 (PMC9104113; doi:10.3390/cancers14092153)
Supplement: Supplementary file 1 [file cancers-14-02153-s001.zip › File S1/original blots-supplementary figures.pdf]

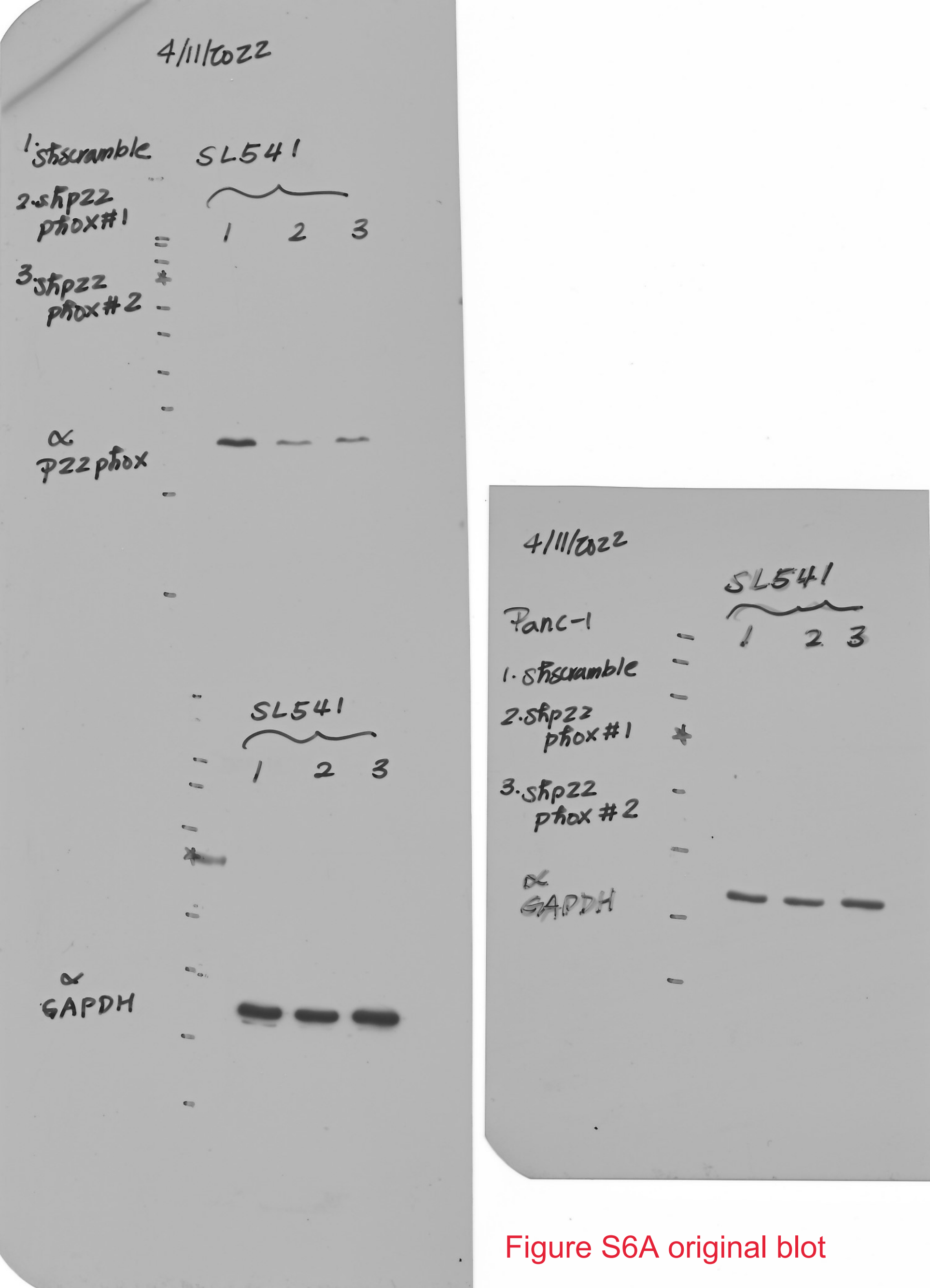

Figure S6A original blot

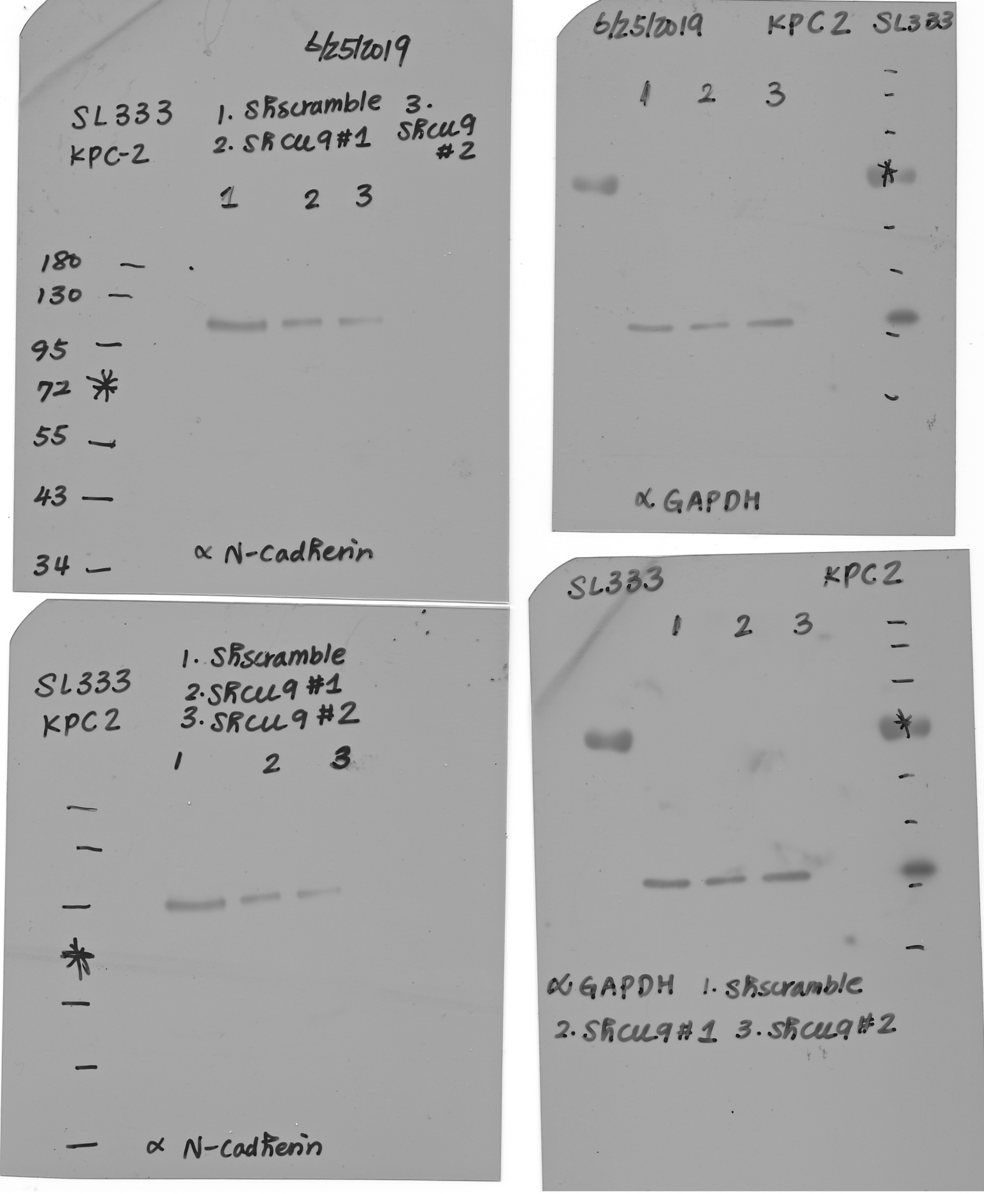

Figure S9 orginal blot
